# Acid–base responsive molecular switching of a [2]rotaxane incorporating two different stations in an axle component†

**DOI:** 10.1039/d4ra03532a

**Published:** 2024-06-19

**Authors:** Risa Yamane, Yuki Asai, Nanami Takiguchi, Ayuna Okamoto, Shintaro Kawano, Yuji Tokunaga, Motohiro Shizuma, Masahiro Muraoka

**Affiliations:** a Department of Applied Chemistry, Faculty of Engineering, Osaka Institute of Technology Asahi-ku Osaka 535-8585 Japan masahiro.muraoka@oit.ac.jp; b Osaka Research Institute of Industrial Science and Technology Joto-ku Osaka 536-8553 Japan; c Department of Materials Science and Engineering, Faculty of Engineering, University of Fukui Bunkyo Fukui 910-8507 Japan

## Abstract

Interlocked compounds such as rotaxanes and catenanes exhibit unique kinetic properties in response to external chemical or physical stimuli and are therefore expected to be applied to molecular machines and molecular sensors. To develop a novel rotaxane for this application, an isophthalamide macrocycle and a neutral phenanthroline axle were used. Stable pseudorotaxanes are known to be formed using hydrogen bonds and π–π interactions. In this study, we designed a non-symmetric axial molecule and synthesized a [2]rotaxane with the aim of introducing two different stations; a phenanthroline and a secondary amine/ammonium unit. Furthermore, ^1^H NMR measurements demonstrated that the obtained rotaxane acts as a molecular switch upon application of external acid/base stimuli.

## Abstract 2

To design new types of rotaxanes for pH-driven molecular switches, we synthesized a [2]rotaxane with a non-symmetric axial molecule with two different stations. This rotaxane acts as a molecular switch upon application of external acid/base stimuli.

## Graphical abstract

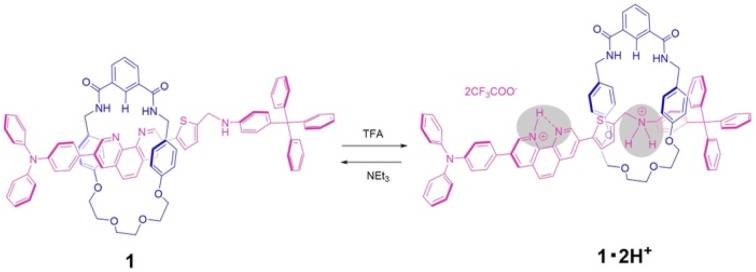

## Introduction

Stimuli-responsive molecular switches are among the most attractive molecular machines^1–4^ in the field of supramolecular chemistry. Rotaxanes (examples of mechanically interlocked molecules, MIMs) are prototypes of molecular switches because they undergo dynamic molecular motion between the macrocycle and axle components.^5–8^ The macrocycle can be translocated at multiple binding sites on the axle component, so-called stations, by controlling different affinities of the macrocycle for external stimuli such as pH,^9–12^ redox,^13–15^ ions,^16^ and lights.^17–19^ Control by protonation and deprotonation is one of the most convenient driving forces to perform molecular switching motions in rotaxanes. However, despite continuous development in pH-driven molecular switches and molecular shuttles, there are only limited examples^9,10,20–22^ of functional molecular skeletons. In addition, many examples revealed that when the ON–OFF discrimination of high affinity host–guest interactions between macrocycle and axle is defective, then the equilibrium distribution of the macrocycle, located with high stabilities on the stations during molecular switching, is ambiguous. Therefore, it would be useful to design new types of rotaxanes for pH-driven molecular switches, which involves the incorporation of new stations as well as translocation between the two stations by external stimuli. The development of a highly efficient and simple synthetic method should be accomplished without the utilization of ammonium (hydrogen bonding) and metal cations to template the formation of rotaxanes such as amide-based rotaxanes.^10,14,20,23^

Previously, we focused on the synthesis of hydrogen-bond templated rotaxanes^24–26^ as well as the incorporation of π–π interactions for the purpose of developing new rotaxanes for molecular switching. We chose a neutral phenanthroline derivative interpenetrated in the cavity of a macrocycle having an isophthalic bisamide skeleton without metal cation templates.^24,25^ We were successful in threading a phenanthroline derivative through an isophthalic bisamide macrocycle with a half dibenzo-crown ether component, which was confirmed by an X-ray analysis of the crystal structure.^25^ In this system, the hydrogen bond between the two amide groups of the macrocycle and the nitrogen of the phenanthroline, as well as π–π and CH–π interactions of phenanthroline with the macrocycle, were observed. Additionally, it was shown that a symmetrical rotaxane can be synthesized with a yield of 60% by efficiently forming a pseudorotaxane from the macrocycle and the phenanthroline axle and then introducing a tritylaniline stopper at both ends. This rotaxane was shown to serve as a reversible pH-controlled molecular switch between two stations on the phenanthroline and the protonated aniline site.^25,26^

**Scheme 1:**
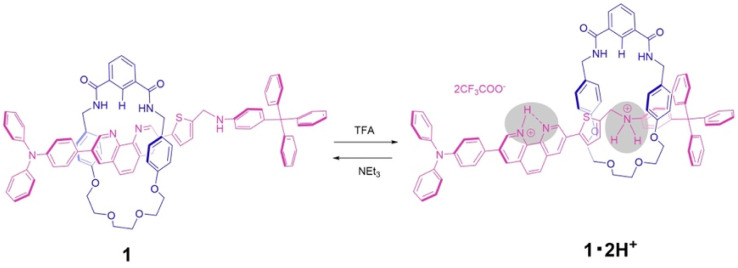
Schematic representation of the acid–base induced molecular switching of [2]rotaxane 1.

Here we report a new type of pH-driven molecular switch based on the phenanthroline station under neutral conditions, and the ammonium station under acidic conditions, as an axle component for an isophthalic bisamide macrocycle with a half dibenzo-crown ether component. This new rotaxane 1 contains the phenanthroline unit within the axle component, which in the neutral form performs as a hydrogen bond-accepting and is constructed by using a hydrogen-bond template synthetic strategy. Upon addition of acid, molecular switching is modulated by the removal from the cavity of a wheel component from the phenanthroline station to the ammonium station of an axle component through the double protonation followed by replacement onto an ethylene glycol moiety of the wheel component. Furthermore, the subsequent addition of base resulted in the deprotonation of the axle component and a return of the wheel component to the phenanthroline station (Scheme 1).

## Experimental

### Synthesis of rotaxane 1

**Scheme 2:**
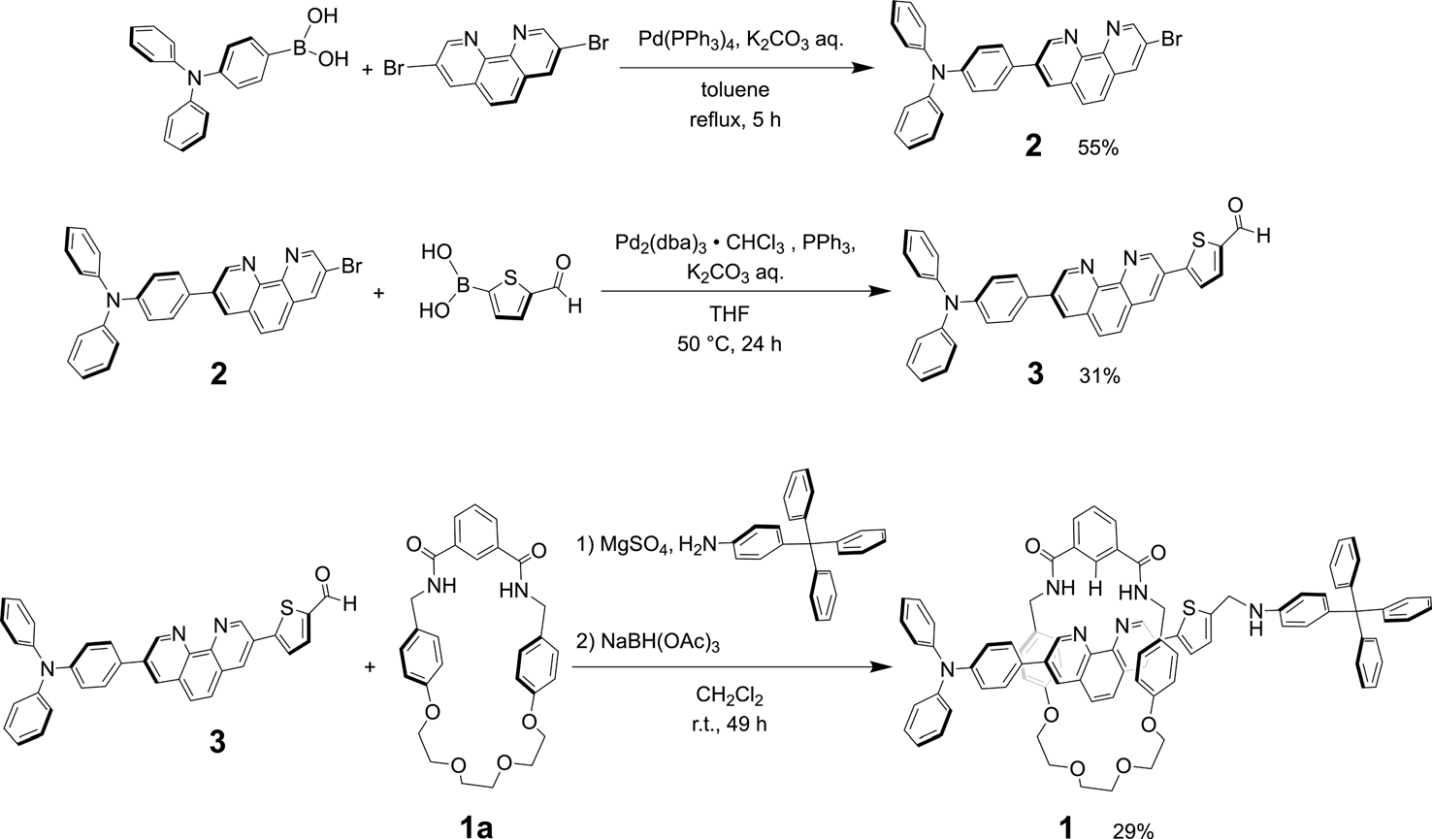
Synthesis of [2]rotaxane 1.

The synthesis of rotaxane 1 can be achieved by reductive amination method of thread precursor 3 with macrocycle 1a^24^ as shown in Scheme 2.

### Product 2

A solution of 3,8-dibromo-1,10-phenanthroline (0.604 g, 1.79 mmol), 4-(diphenylamino)phenylboronic acid (0.521 g, 1.80 mmol), Pd(PPh_3_)_4_ (0.0585 g, 0.0506 mmol) and K_2_CO_3_ (1.50 g, 10.9 mmol) in toluene (100 mL) and distilled water (15 mL) was refluxed and stirred at 110 °C for 5 h under a nitrogen atmosphere. The product was extracted with dichloromethane, washed with water, dried over anhydrous sodium sulfate, and concentrated by vacuum evaporation after filtration. The crude product obtained as an orange solid was purified by open silica gel column chromatography (hexane : dichloromethane = 1 : 1, dichloromethane, and dichloromethane : methanol = 99 : 1–95 : 5). Purification was again conducted by medium-pressure column chromatography (silica gel, hexane : dichloromethane = 5 : 5, 3 : 7, and 1 : 9, dichloromethane, and dichloromethane : methanol = 99 : 1–95 : 5). After evaporating the solvent, the product 2 was obtained as an orange solid (496 mg, 55%).

Mp 188–189 °C; ^1^H NMR (400 MHz, CDCl_3_): *δ* (ppm) 9.41 (d, 1H, *J* = 2.4 Hz), 9.18 (d, 1H, *J* = 2.4 Hz), 8.40 (d, 1H, *J* = 2.4 Hz), 8.34 (d, 1H, *J* = 2.4 Hz), 7.87 (d, 1H, *J* = 8.4 Hz), 7.72 (d, 1H, *J* = 8.4 Hz), 7.65 (d, 2H, *J* = 8.4 Hz), 7.31 (m, 4H), 7.22 (d, 2H, *J* = 8.4 Hz), 7.18 (dd, 4H, *J* = 1.2, 8.4 Hz), 7.09 (t, 2H, *J* = 1.2, 7.6 Hz); ^13^C NMR (100 MHz, CDCl_3_): *δ* (ppm) 151.2, 149.6, 148.5, 147.4, 144.43, 144.40, 137.5, 135.7, 132.5, 130.5, 129.5, 128.6, 128.24, 128.19, 125.8, 124.9, 123.6, 123.5, 119.6; HRMS: found *m*/*z* 502.0894, calcd *m*/*z* 502.0913 for [M + H]^+^ (M: C_30_H_20_BrN_3_).

### Thread precursor 3

A solution of 2 (0.203 g, 0.404 mmol), 5-formyl-2-thiopheneboronic acid (0.192 g, 1.23 mmol), Pd_2_(dba)_3_·CHCl_3_ (0.0870 g, 0.0840 mmol), PPh_3_ (0.0854 g, 0.326 mmol) and K_2_CO_3_ (0.351 g, 2.54 mmol) in THF (12.6 mL) and distilled water (2.0 mL) was stirred at 50 °C for 24 h under a nitrogen atmosphere. After removal of THF, the product was extracted using dichloromethane, washed with water, dried over anhydrous sodium sulfate, and concentrated by vacuum evaporation after filtration. The crude product obtained as an orange solid was purified by open alumina column chromatography (dichloromethane and then dichloromethane : methanol = 97 : 3–93 : 7). Purification was again conducted by open silica gel column chromatography (dichloromethane and dichloromethane : methanol = 99.5 : 0.5–95 : 5). After evaporating the solvent, thread precursor 3 was obtained as an orange solid (65.7 mg, 31%).

Mp 244–245 °C; ^1^H NMR (400 MHz, CDCl_3_): *δ* (ppm) 9.97 (s, 1H), 9.48 (d, 1H, *J* = 2.4 Hz), 9.44 (d, 1H, *J* = 2.4 Hz), 8.47 (d, 1H, *J* = 2.4 Hz), 8.37 (d, 1H, *J* = 2.4 Hz), 7.92–7.86 (m, 3H), 7.70 (d, 1H, *J* = 4.0 Hz), 7.67 (d, 2H, *J* = 8.4 Hz), 7.31 (m, 4H), 7.23 (d, 2H, *J* = 8.4 Hz), 7.18 (dd, 4H, *J* = 1.2, 8.4 Hz), 7.09 (t, 2H, *J* = 1.2, 7.6 Hz); ^13^C NMR (100 MHz, CDCl_3_): *δ* (ppm) 182.8, 145.0, 149.7, 148.6, 147.7, 147.4, 146.2, 144.4, 143.8, 137.5, 135.8, 132.8, 132.5, 130.4, 129.6, 129.1, 128.3, 128.13, 128.07, 128.0, 126.8, 125.7, 125.0, 123.6, 123.5; HRMS: found *m*/*z* 534.1617, calcd *m*/*z* 534.1635 for [M + H]^+^ (M: C_35_H_23_N_3_O_1_S_1_).

### Rotaxane 1

A solution of macrocycle 1a (0.0755 g, 0.155 mmol), thread precursor 3 (0.0684 g, 0.128 mmol) and 4-tritylaniline (0.0546 g, 0.163 mmol) in commercially available super dehydrated dichloromethane (FUJIFILM Wako Pure Chemical Corporation) (4.1 mL) containing MgSO_4_ as a dehydrating agent was stirred for 22 h at room temperature under a nitrogen atmosphere. Sodium triacetoxyborohydride (0.177 g, 0.797 mmol) was added and the mixture was stirred for an additional 27 h. The product was extracted with dichloromethane, washed with water two times, dried over anhydrous sodium sulfate, and concentrated by vacuum evaporation after filtration. The crude product obtained as an orange solid was purified by open silica gel column chromatography (hexane : dichloromethane = 1 : 1, dichloromethane, and dichloromethane : methanol = 99.5 : 0.5–93 : 7). Purification was again conducted by medium-pressure column chromatography (silica gel, dichloromethane : methanol = 95 : 5). After evaporating the solvent, rotaxane 1 was obtained as a yellow solid (41.8 mg, 29%). Although the unreacted macrocycle could be recovered in 22% yield, the dumbbell 4 ^27^ was not isolated, probably due to decomposition of the thread component 3 during the reaction.

Mp 178–180 °C; ^1^H NMR (600 MHz, CDCl_3_): *δ* (ppm) 10.2 (s, 1H), 8.77 (d, 1H, *J* = 2.4 Hz), 8.66 (d, 1H, *J* = 2.4 Hz), 8.46 (d, 1H, *J* = 2.4 Hz), 8.44 (d, 1H, *J* = 2.4 Hz), 8.41 (dd, 2H, *J* = 1.2, 7.8 Hz), 8.14 (t, 2H, *J* = 4.5 Hz), 7.89 (d, 1H, *J* = 8.4 Hz), 7.87 (d, 1H, *J* = 8.4 Hz), 7.69–7.66 (m, 3H), 7.43 (d, 1H, *J* = 3.6 Hz), 7.32 (t, 4H, *J* = 7.8 Hz), 7.24–7.15 (m, 21H), 7.09 (t, 3H, *J* = 6.9 Hz), 7.04 (d, 2H, *J* = 8.7 Hz), 6.64 (d, 2H, *J* = 8.7 Hz), 6.02 (d, 4H, *J* = 8.7 Hz), 5.64 (d, 4H, *J* = 8.7 Hz), 4.58 (s, 2H), 4.14 (dd, 2H, *J* = 5.4, 14.4 Hz), 3.96–3.92 (m, 6H), 3.89–3.72 (m, 8H); ^13^C NMR (150 MHz, CDCl_3_): *δ* (ppm) 166.3, 156.4, 148.4, 147.6, 147.5, 147.3, 146.9, 146.3, 145.5, 145.0, 144.1, 144.0, 139.7, 136.8, 134.7, 133.9, 133.0, 132.2, 131.9, 131.2, 130.7, 129.6, 129.1, 128.9, 128.8, 128.7, 128.1, 128.0, 127.9, 127.7, 127.5, 126.5, 126.1, 125.9, 125.0, 124.7, 123.7, 123.6, 120.4, 112.4, 112.3, 71.3, 70.3, 66.8, 64.4, 44.3, 44.1; HRMS: found *m*/*z* 672.2745, calcd *m*/*z* 672.2768 for [M + 2H]^2+^ (M: C_88_H_74_N_6_O_6_S_1_).

## Results and discussion

When Pd_2_(dba)_3_·CHCl_3_ was used as a catalyst with 2-dicyclohexylphosphino-2′,6′-dimethoxybiphenyl (SPhos) or triphenylphosphine (PPh_3_) as ligands in the Suzuki coupling reaction with 3,8-dibromophenanthroline and 4-(diphenylamino)phenylboronic acid, monosubstituted product 2 could not be isolated.^28^ The reactivity of product 2 on this catalyst seemed to be very high, and the disubstituted byproduct was obtained as the main component. On the other hand, as shown in Scheme 2, when Pd(PPh_3_)_4_ was used as a catalyst, the target monosubstituted product 2 was obtained in a yield of 55%. For the synthesis of thread precursor 3, 5-formyl-2-thiopheneboronic acid as a coupling reagent and the obtained thread precursor 3 was found to be unstable under reflux conditions. Therefore, the coupling reaction was performed at 50 °C to improve the reaction yield (31%) after purification by alumina followed by silica gel column chromatography.

**Fig. 1:**
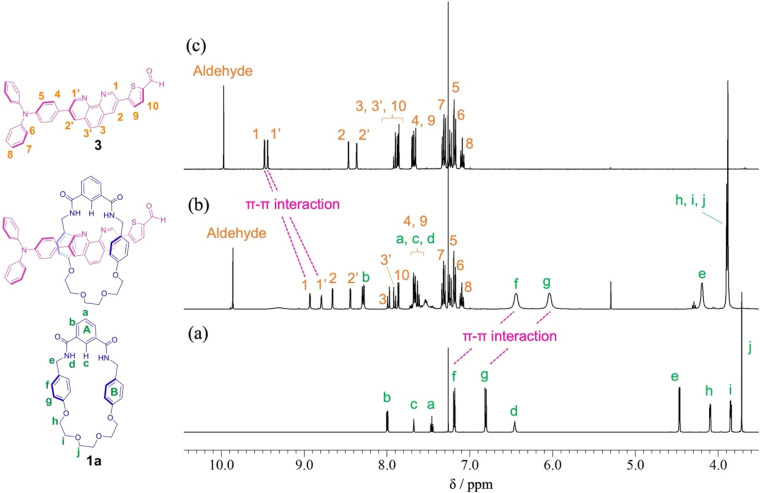
Partial ^1^H NMR spectra (400 MHz, CDCl_3_) of (a) macrocycle 1a, (b) pseudo[2]rotaxane, and (c) thread precursor 3.

[2]Rotaxane 1 was prepared *via* a hydrogen-bond template stoppering methodology. Equimolar amounts of thread precursor 3 and macrocycle 1a were dissolved in CDCl_3_. As shown in Fig. 1, ^1^H NMR measurements were performed to ensure the formation of the pseudorotaxane, since the ratio of the respective integrals of macrocycle 1a and thread precursor 3 is observed to indicate the formation of a 1 : 1 complex. Protons (1 and 1′) of the phenanthroline site and protons f and g of aromatic ring B of the macrocycle were detected with large upfield shifts due to complex formation by π–π interactions (Δ*δ* 0.6 ppm for proton 1 and Δ*δ* 0.7 ppm for proton 1′, and Δ*δ* 0.7 ppm for protons f and g). The binding constant (*K*_a_) for the complexation were measured by ^1^H NMR titration experiments.^29^ Monitoring the upfield shifts of protons f and g upon adding 3 allowed us to estimate an association constant of 1.2 ± 0.08 × 10^3^ as shown in Fig. S22.†

**Fig. 2:**
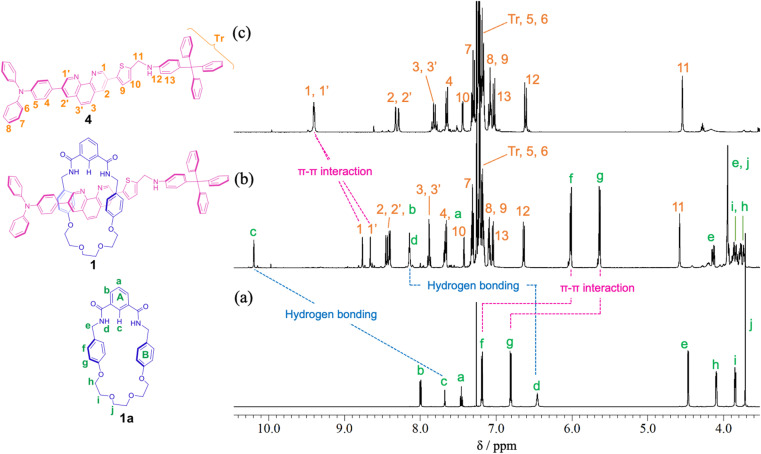
Partial ^1^H NMR spectra (400 MHz, CDCl_3_) of (a) macrocycle 1a, (b) [2]rotaxane 1, and (c) dumbbell 4.

For the synthesis of [2]rotaxane 1, thread precursor 3 and macrocycle 1a were dissolved in CH_2_Cl_2_, and then 1.3 equivalents of 4-tritylaniline as the stopper site and MgSO_4_ as a dehydrating agent were added to the solution followed by the addition of NaBH(OAc)_3_ as a reducing agent (Scheme 2). After purification, ^1^H NMR measurements were performed, showing that signals due to protons d of two benzamide groups (Δ*δ* 1.7 ppm) and proton c of aromatic ring A of the wheel component (Δ*δ* 2.5 ppm) were shifted significantly downfield, indicating hydrogen bonds between both components as shown in Fig. 2. In addition, signals due to protons f and g of aromatic rings B of the wheel component (Δ*δ* 1.2 ppm) were shifted more upfield than for the [2]pseudorotaxane structure. Therefore, [2]rotaxane 1 was formed by hydrogen bonds between the two benzamide groups and proton c of aromatic ring A of the wheel component and the nitrogens of the phenanthroline site of the axle component, and by the π–π interaction between phenanthroline site of the axle component and aromatic ring B of the wheel component (Fig. 2).

In the ROESY spectrum of [2]rotaxane 1, correlations were observed between protons g of aromatic ring B of the wheel component and protons 3 and 3′ of phenanthroline site of the axle component, protons d of two benzamide groups of the wheel component and protons 1 and 1′ of phenanthroline site of the axle component, and proton c of aromatic ring A of the wheel component and protons 1 and 1′ of phenanthroline site of the axle component, indicating that the wheel component is located at phenanthroline site of the axle component (Fig. S11†). When rotaxane 1 solution in CDCl_3_ was cooled from room temperature to −60 °C, there was no change at all in the ^1^H NMR spectrum, indicating that the phenanthroline moiety in the axle component of rotaxane 1 functions as a station for the wheel component under neutral conditions. The result is also supported by the prediction that the p*K*_a_ of the phenanthroline moiety is higher than that of the aniline moiety. Therefore, we introduced an aniline moiety in the structure of rotaxane 1 that can function as the second station under acidic conditions for the designing of a molecular switching system. Since the ammonium ion of the protonated aniline moiety has a more localized charge and lower p*K*_a_ than that of the protonated phenanthroline moiety along with the larger number of hydrogen bonds, the aniline moiety can considerably function as a second station by the electrostatic interaction and hydrogen bonding between the ammonium ion and the oxyethylene moiety of the wheel component even under the double protonation.

**Fig. 3:**
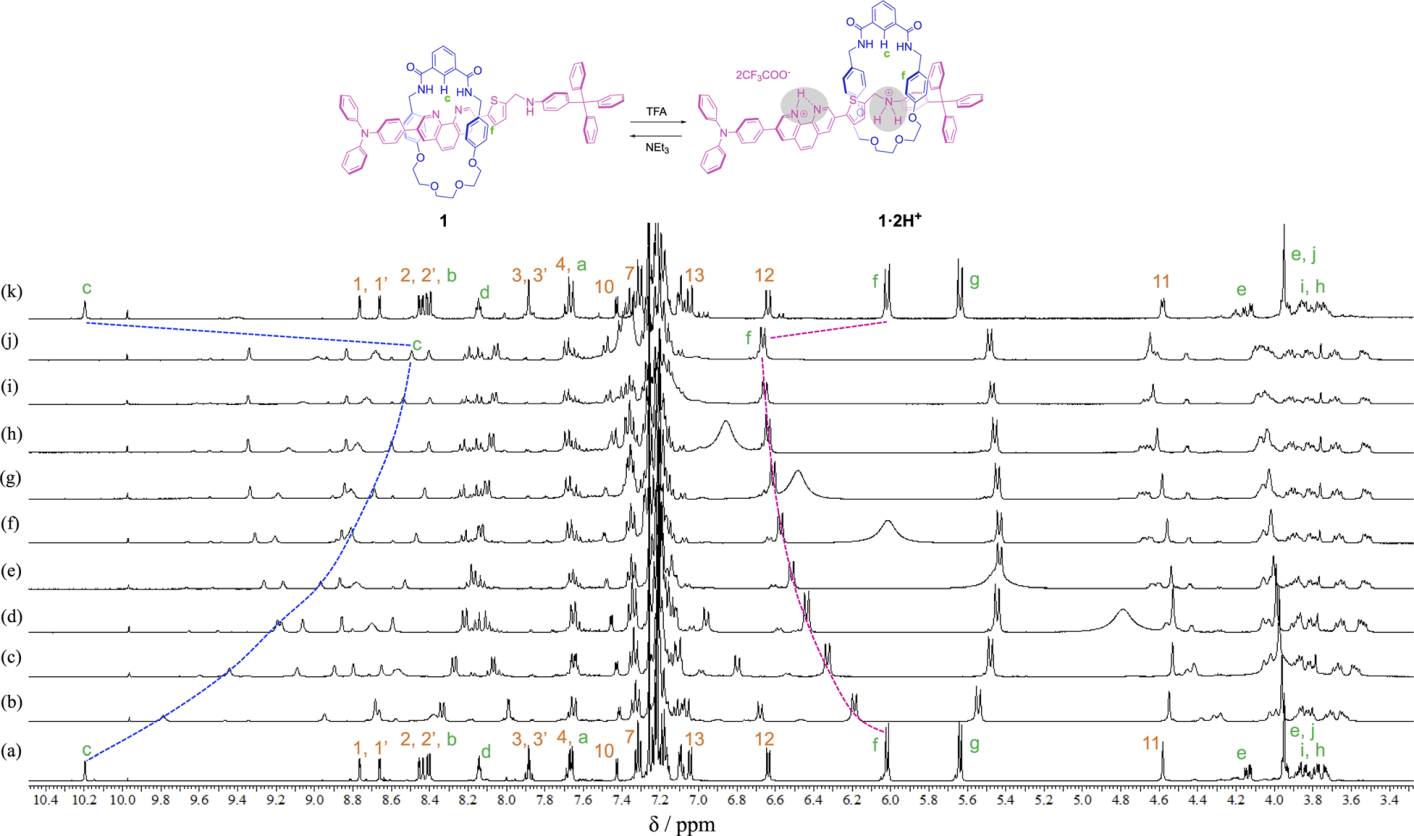
Partial ^1^H NMR spectra (400 MHz, CDCl_3_) of (a) rotaxane 1, (b)–(j) the mixture obtained after adding TFA (1, 2, 3, 4, 5, 6, 7, 8, and 9 eq. respectively) to rotaxane 1 solution, and (k) the mixture obtained after adding NEt_3_ (9 eq.) to the mixture obtained by addition of TFA (9 eq.) to rotaxane 1.

In order to observe the acid–base responsive molecular switching of [2]rotaxane 1, trifluoroacetic acid (TFA) as an acid and triethylamine (NEt_3_) as a base were used as stimuli. As shown in Fig. 3, when excess amounts of TFA (9 equivalents) were added to the synthesized [2]rotaxane 1, the ^1^H NMR spectra indicated that the protonation of [2]rotaxane 1 dissociated the hydrogen bond between amide NH– and phenanthroline, the signals of protons c and f shifted up- and downfield, respectively. These observations suggested a loss of the deshielding and shielding effects of the phenanthroline moiety. However, the signal due to proton g did not shift and signals associated with the oxyethylene moieties in the wheel component were observed to split due to the discrimination of each of the geminal protons of the oxyethylene chain in the wheel component. Thus, it is considered that migration of the wheel component occurred toward the anilinium station of the protonated axle component, indicating that the oxyethylene site and the anilinium site interacted with each other by hydrogen bonding.

**Fig. 4:**
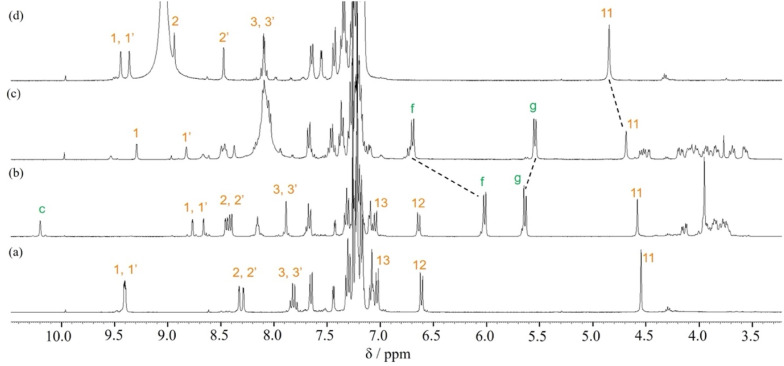
Partial ^1^H NMR spectra (400 MHz, CDCl_3_) of (a) dumbbell 4, (b) rotaxane 1, (c) rotaxane 1·2H^+^ obtained after adding TFA (15 eq.) to rotaxane 1, and (d) dumbbell 4·2H^+^ obtained after adding TFA (15 eq.) to dumbbell 4.

In order to confirm that the wheel component of [2]rotaxane 1·2H^+^ resided on the anilinium site of the axle component in response to treatment with TFA, excess amounts of TFA (15 equivalents) were added to dumbbell 4 in CDCl_3_ and the ^1^H NMR spectrum was compared with that for [2]rotaxane 1·2H^+^, and showed that the signal due to proton 11 of [2]rotaxane 1·2H^+^ was shifted slightly upfield (*ca.* 0.1 ppm) relative to proton 11 of dumbbell 4·2H^+^ (Fig. 4). This indicates that aromatic shielding is observed for the dumbbell proton 11 adjacent to the anilinium unit and wheel benzyl protons f and g.

To neutralize the protonated rotaxane 1·2H^+^ after addition of 9 equivalents of TFA, when 9 equivalents of NEt_3_ were added as a base, the ^1^H NMR signals returned to the original positions for neutral [2]rotaxane 1 (Fig. 3), confirming that the [2]rotaxane 1 functioned as a molecular switch. Such molecular switching by addition of acid and base was confirmed to occur repeatedly (see Fig. S23 and S24†).

## Conclusions

In this study, we introduced a heterologous stopper moiety into a rotaxane by reductive amination of a terminal formyl group to obtain the desired unsymmetric structure. Addition of acid as an external stimulus to the rotaxane resulted in double protonation of the nitrogens of the phenanthroline and the aniline, shifting the wheel component to the anilinium station. The subsequent addition of base resulted in the deprotonation of the axle component and a return of the wheel component to the phenanthroline station, as observed by ^1^H NMR spectroscopy. These pH-responsive rotaxanes are expected to be used for molecular machines and molecular sensors in the future.

## Data availability

The data supporting this article have been included as part of the ESI.†

## Conflicts of interest

There are no conflicts to declare.

## Supplementary Material

RA-014-D4RA03532A-s001
