# Supplementary material for: Acid–base responsive molecular switching of a [2]rotaxane incorporating two different stations in an axle component
Source: RSC Adv. 2024 Jun 19;14(28):19780–6. doi: 10.1039/d4ra03532a (PMC11188621; doi:10.1039/d4ra03532a)
Supplement: RA-014-D4RA03532A-s001 [file RA-014-D4RA03532A-s001.pdf]

Electronic Supplementary Material (ESI) for RSC Advances.
This journal is © The Royal Society of Chemistry 2024

Supplementary Information

Acid-Base Responsive Molecular Switching of a [2]Rotaxane Incorporating Two Different Stations in an Axle Component

Risa Yamane,[a] Yuki Asai,[a] Nanami Takiguchi,[a] Ayuna Okamoto,[a] Shintaro Kawano,[b] Yuji Tokunaga,[c] Motohiro Shizuma,[b] and Masahiro Muraoka*[a]

[a]Department of Applied Chemistry, Faculty of Engineering, Osaka Institute of Technology, Asahi-ku, Osaka 535-8585, Japan.

[b]Osaka Research Institute of Industrial Science and Technology, Joto-ku, Osaka 536-8553, Japan.

[c]Department of Materials Science and Engineering, Faculty of Engineering, University of Fukui, Bunkyo, Fukui 910-8507, Japan.

1. General

Melting points (mp) were measured on a Yanaco MP-J3 instrument. The ^{1}H and ^{13}C NMR measurements were carried out with JEOL JNM-ECZ400S (400 MHz) and JNM-ECA600 (600 MHz) instruments. The NMR chemical shifts are reported in ppm with reference to residual protons and carbons of $CDCl_3$ (δ = 7.26 ppm for ^{1}H NMR; δ = 77.00 ppm for ^{13}C NMR). UV-vis absorption spectra were measured with a Shimadzu UV-2450 spectrometer. Spectrophotometer were measured with a Shimadzu RF-5300PC spectrometer.

4-(Diphenylamino)phenylboronic acid, 5-formyl-2-thiopheneboronic acid (contains varying amounts of Anhydride) and sodium triacetoxyborohydride were purchased from Tokyo Chemical Industry Co., Ltd. Potassium carbonate, super dehydrated toluene, distilled water, dichloromethane, triphenyl phosphine, super dehydrated tetrahydrofuran, super dehydrated dichloromethane, sodium sulfate (anhydrous), magnesium sulfate (anhydrous), triethylamine and trifluoroacetic acid were purchased from Wako Pure Chemical Industries, Ltd. 4-Tritylaniline were purchased from Alfa Aesar A Jobson Matthey, Ltd.

Thin layer chromatography (TLC) was performed on glass plates coated with 0.25 mm thick silica gel 60F-254 (Merck). Column chromatography was performed using PSQ 100B (Fuji Silysia). Medium pressure silica gel column chromatography was performed using PSQ 60B (Fuji Silysia). Alumina column chromatography was performed using Activated Alumina about 75μm (Wako Pure Chemical Industries, Ltd.).

All reactions were carried out under a nitrogen atmosphere.

2. Synthesis of dumbbell 4

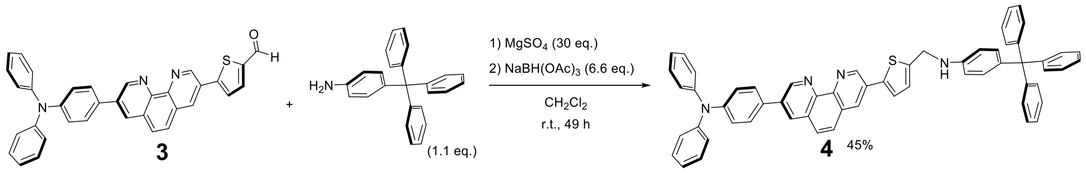

A solution of thread precursor **3** (0.0608 g, 0.114 mmol) and 4-tritylaniline (0.0455 g, 0.128 mmol) in super dehydrated dichloromethane (4.0 ml) containing $MgSO_4$ as a dehydrating agent was stirred for 21 hours at room temperature under a nitrogen atmosphere. Sodium triacetoxyborohydride (0.1676 g, 0.755 mmol) was added and the mixture was stirred for a further 41 hours. The product was extracted by dichloromethane, washed with water tow times, dried over anhydrous sodium sulfate, and concentrated by vacuum evaporator after filtration. The crude product obtained as an orange solid was purified by open silica gel column chromatography using dichloromethane, and dichloromethane : methanol = 99.9:0.1-91:9 as eluents. Purification was again conducted by medium pressure column chromatography (silica gel, dichloromethane, and dichloromethane : methanol = 99.9:0.1-93:7 as eluents). After evaporating the solvent, the product dumbbell **4** was obtained as an orange solid. (35.9 mg, 45%)

M.p. 262-263 °C; ^{1}H NMR (400 MHz, $CDCl_3$): δ (ppm) 9.40 (dd, 2H, J = 2.0Hz), 8.33 (d, 1H, J = 1.6 Hz), 8.29 (d, 1H, J = 1.6 Hz), 7.82 (q, 2H, J = 9.2 Hz), 7.65 (d, 2H, J = 8.8 Hz), 7.44 (d, 1H, J = 3.6 Hz), 7.31 (t, 4H, J = 8.0 Hz), 7.24-7.15 (m, 21H), 7.10-7.01 (m, 3H), 7.02 (d, 2H, J = 2.0 Hz), 6.61 (d, 2H, J = 8.8 Hz), 4.54 (s, 2H); ^{13}C NMR (150 MHz, $CDCl_3$): δ (ppm) 149.5, 148.5, 147.9, 147.5, 147.3, 145.4, 145.1, 145.1, 144.8, 139.6, 136.9, 135.4, 132.6, 132.2, 131.3, 130.9, 129.6, 128.7, 128.5, 128.3, 127.7, 127.5, 126.9, 126.4, 126.1, 125.9, 125.0, 124.8, 123.7, 123.6, 120.4, 64.4, 44.1 ; HRMS: Found *m/z* 853.3381, calcd. *m/z* 853.3359 for $[M + H]^+$ (M: $C_{60}H_{44}N_4S$).

3. NMR Spectra

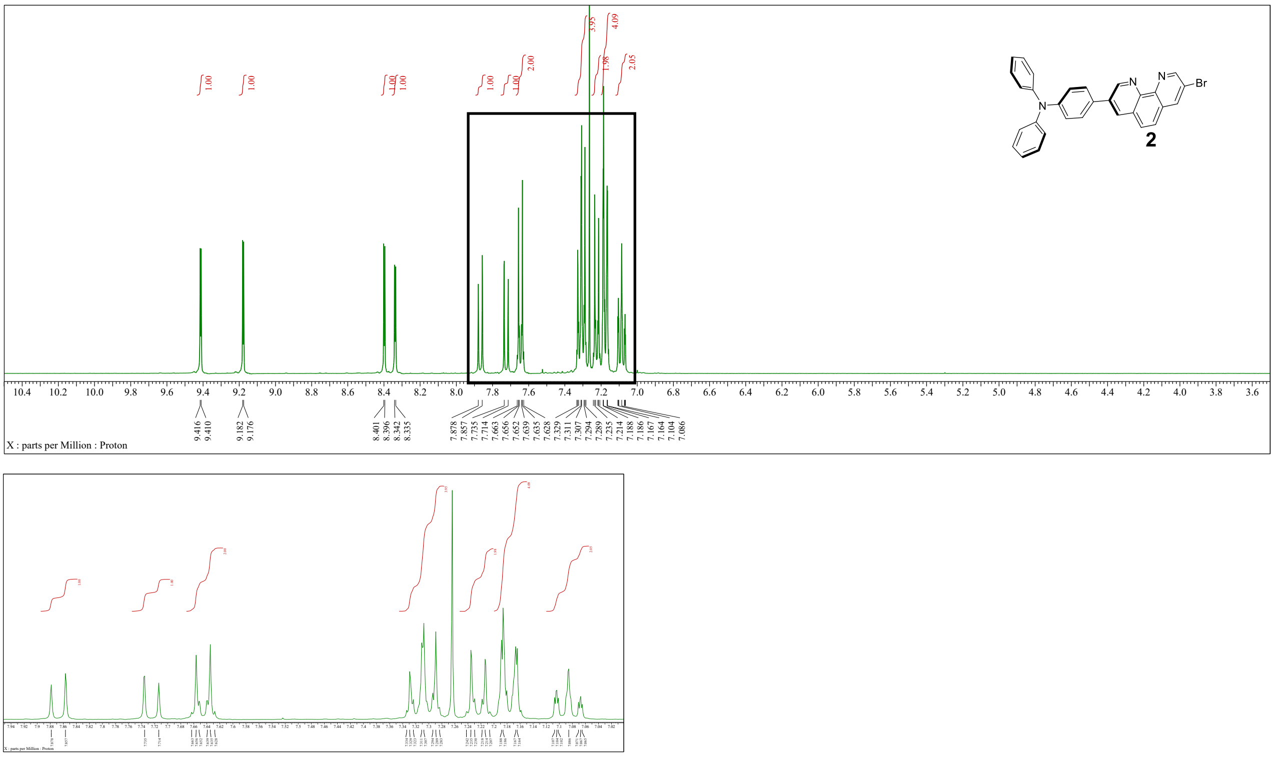

Figure S1. ^{1}H NMR (400 MHz, $CDCl_3$) spectrum of 4-(8-Bromo-1,10-phenanthrolin-3-yl)-*N,N*-diphenylaniline (**2**).

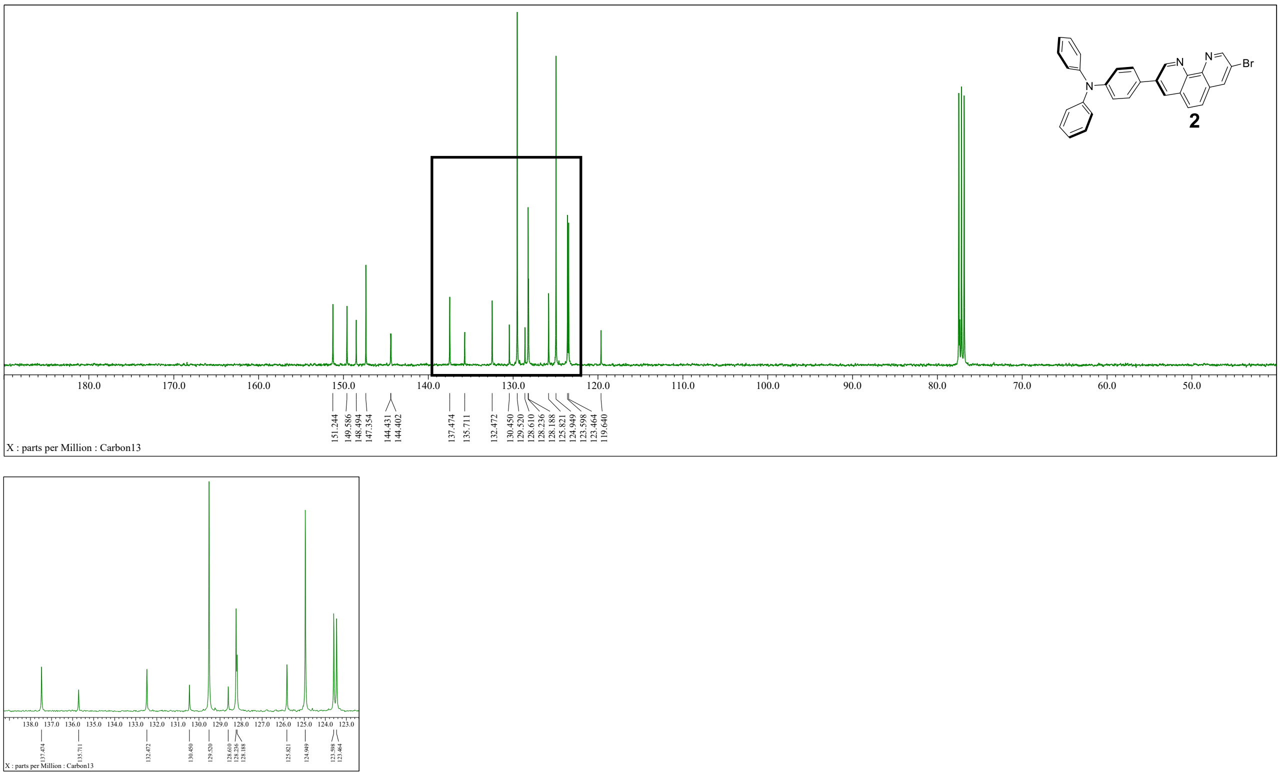

Figure S2. ^{13}C NMR (100 MHz, $CDCl_3$) spectrum of 4-(8-Bromo-1,10-phenanthrolin-3-yl)-*N,N*-diphenylaniline (**2**).

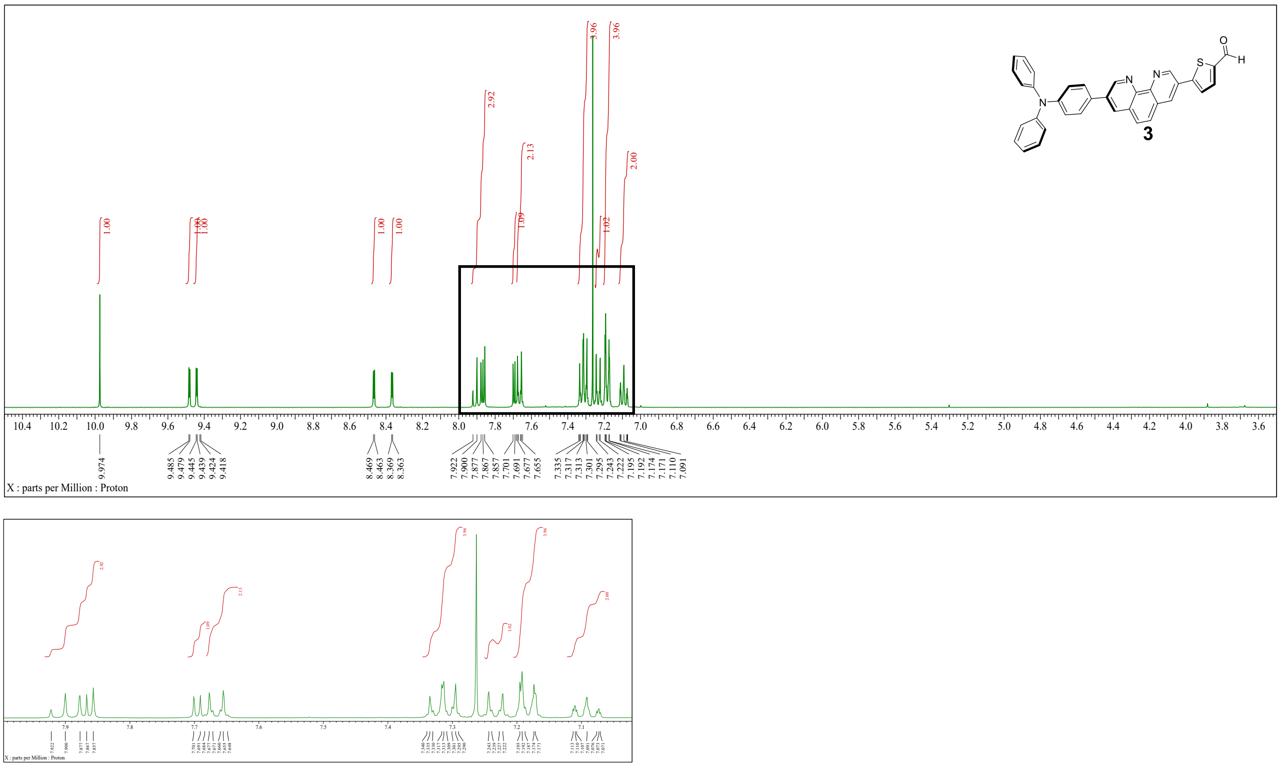

Figure S3. ^{1}H NMR (400 MHz, $CDCl_3$) spectrum of 5-(8-(4-(diphenylamino)phenyl)-1,10-phenanthrolin-3-yl)thiophene-2-carbaldehyde (**3**).

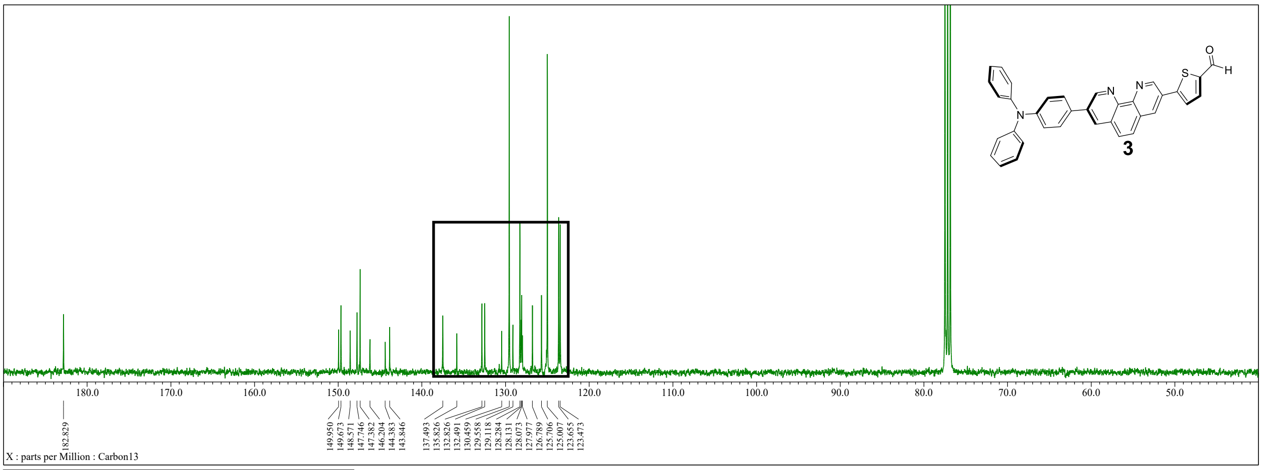

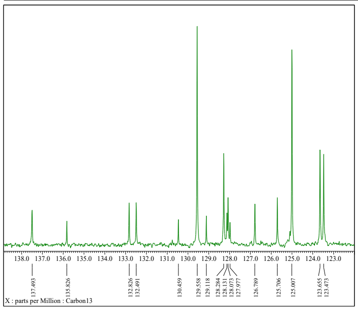

Figure S4. ^{13}C NMR (100 MHz, $CDCl_3$) spectrum of 5-(8-(4-(diphenylamino)phenyl)-1,10-phenanthrolin-3-yl)thiophene-2-carbaldehyde (**3**)

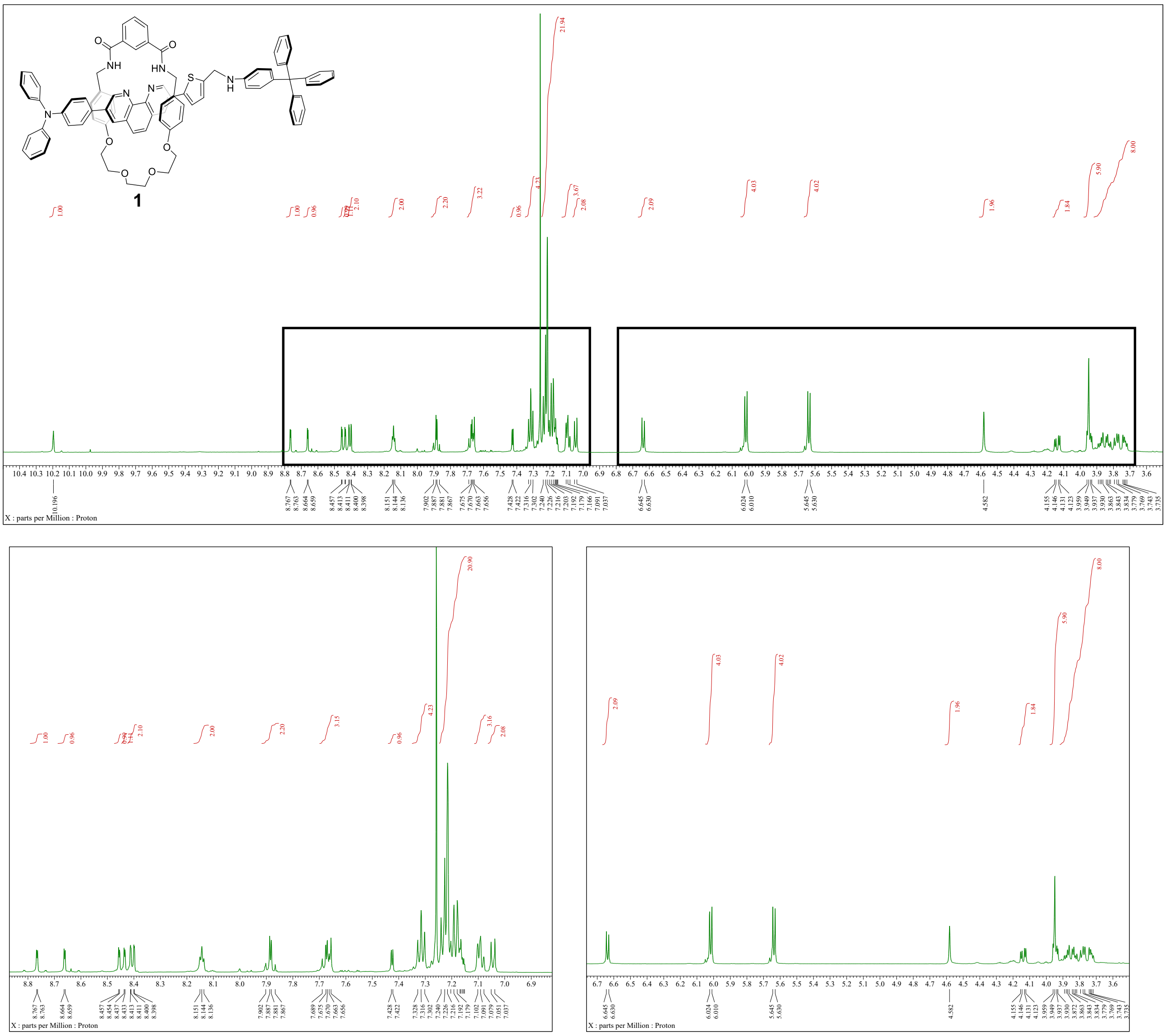

Figure S5. ^{1}H NMR (600 MHz, $CDCl_3$) spectrum of rotaxane **1**.

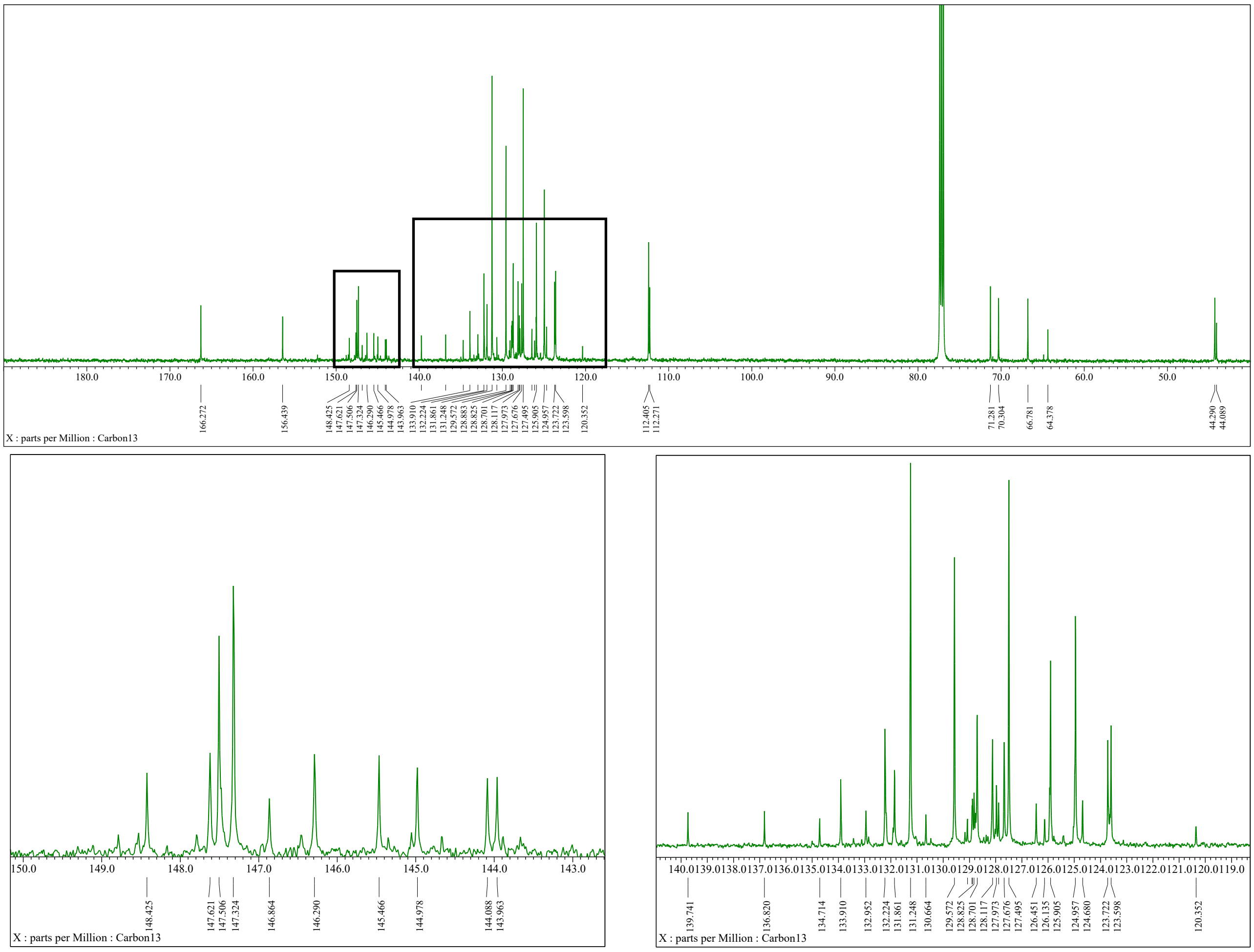

Figure S6. ^{13}C NMR (150 MHz, $CDCl_3$) spectrum of rotaxane **1**.

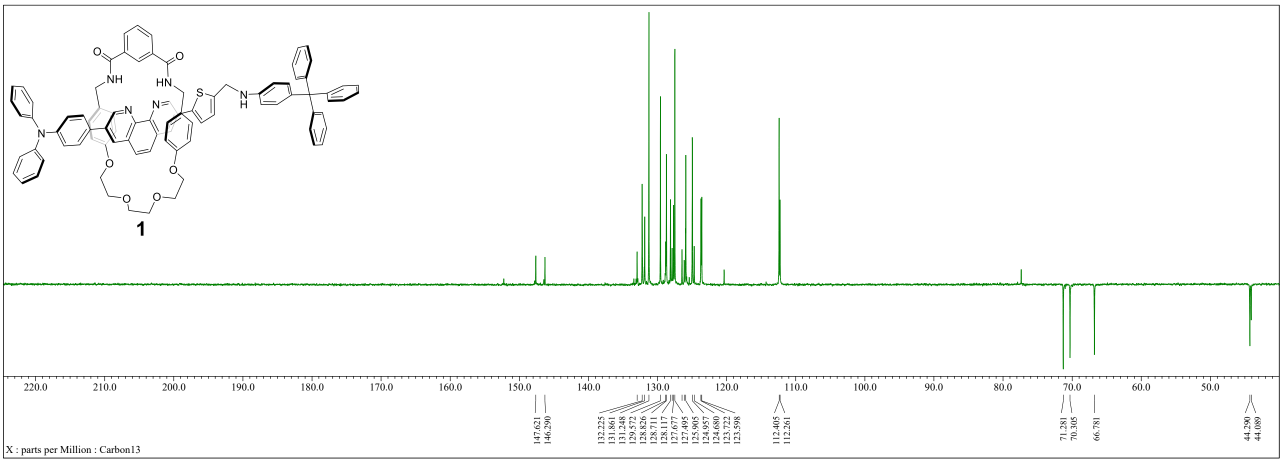

Figure S7. DEPT 135° (150 MHz, $CDCl_3$) spectrum of rotaxane **1**.

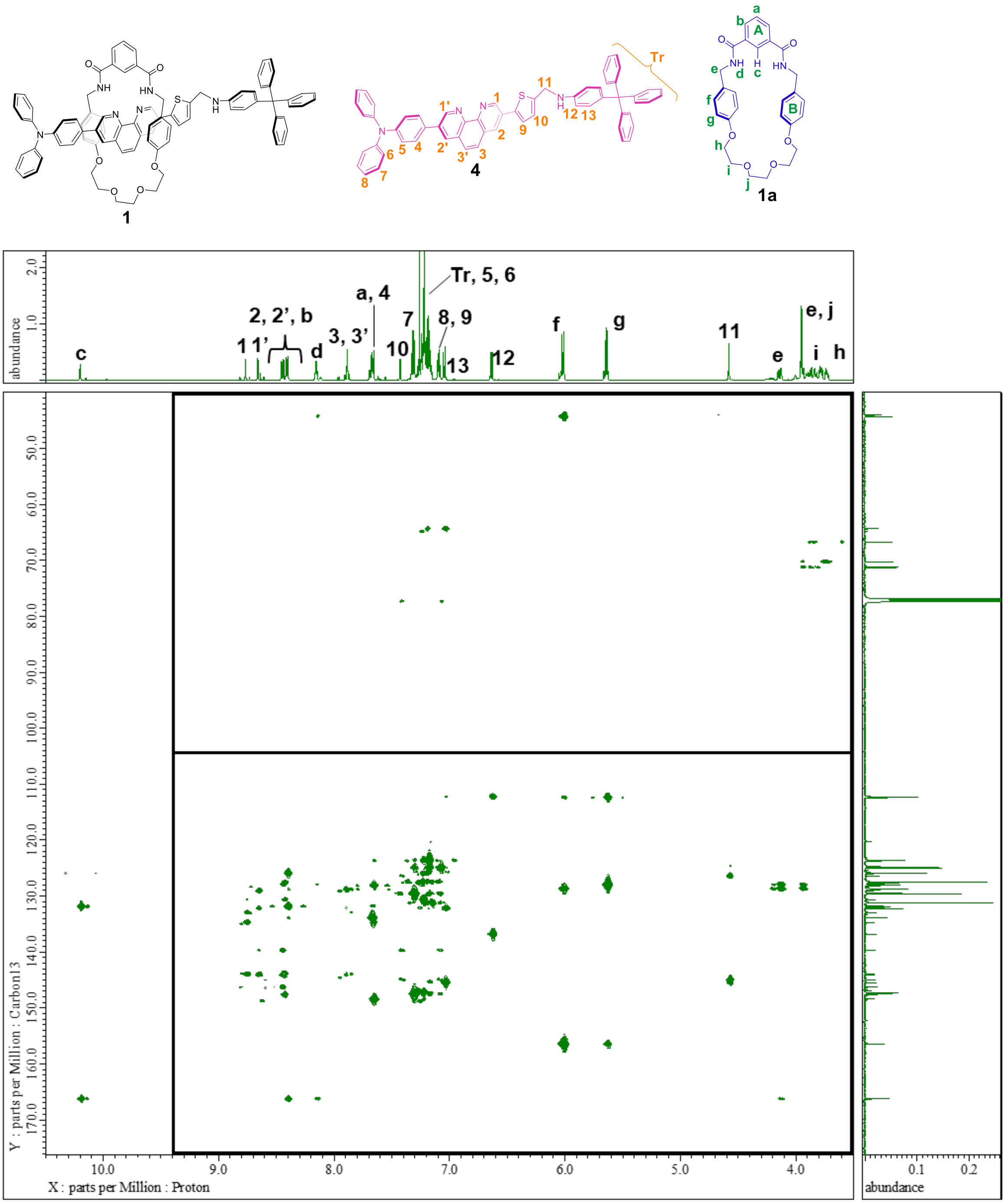

Figure S8-1. HMBC (600 MHz, $CDCl_3$) spectrum of rotaxane **1**.

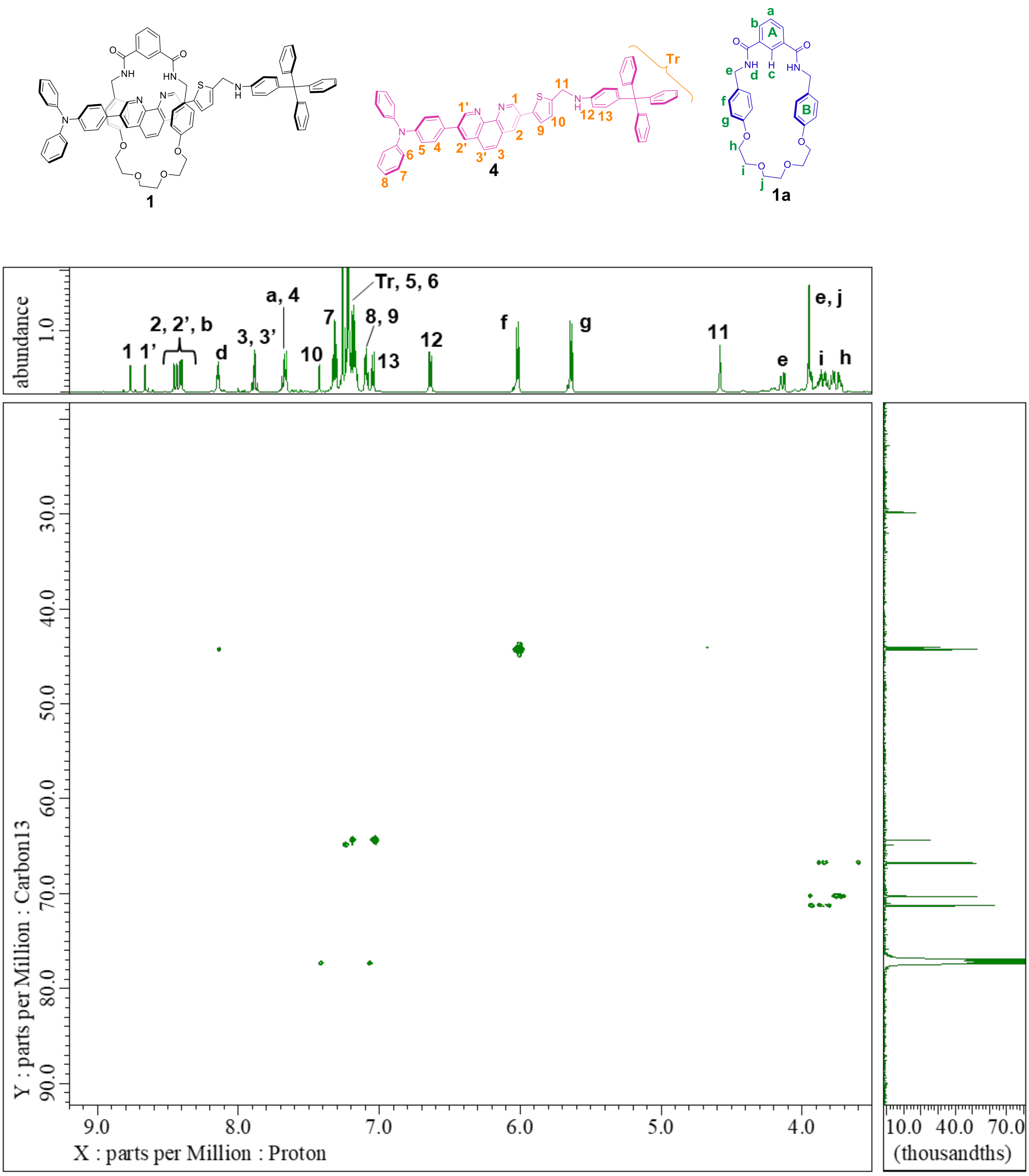

Figure S8-2. HMBC (600 MHz, $CDCl_3$) spectrum of rotaxane **1**.

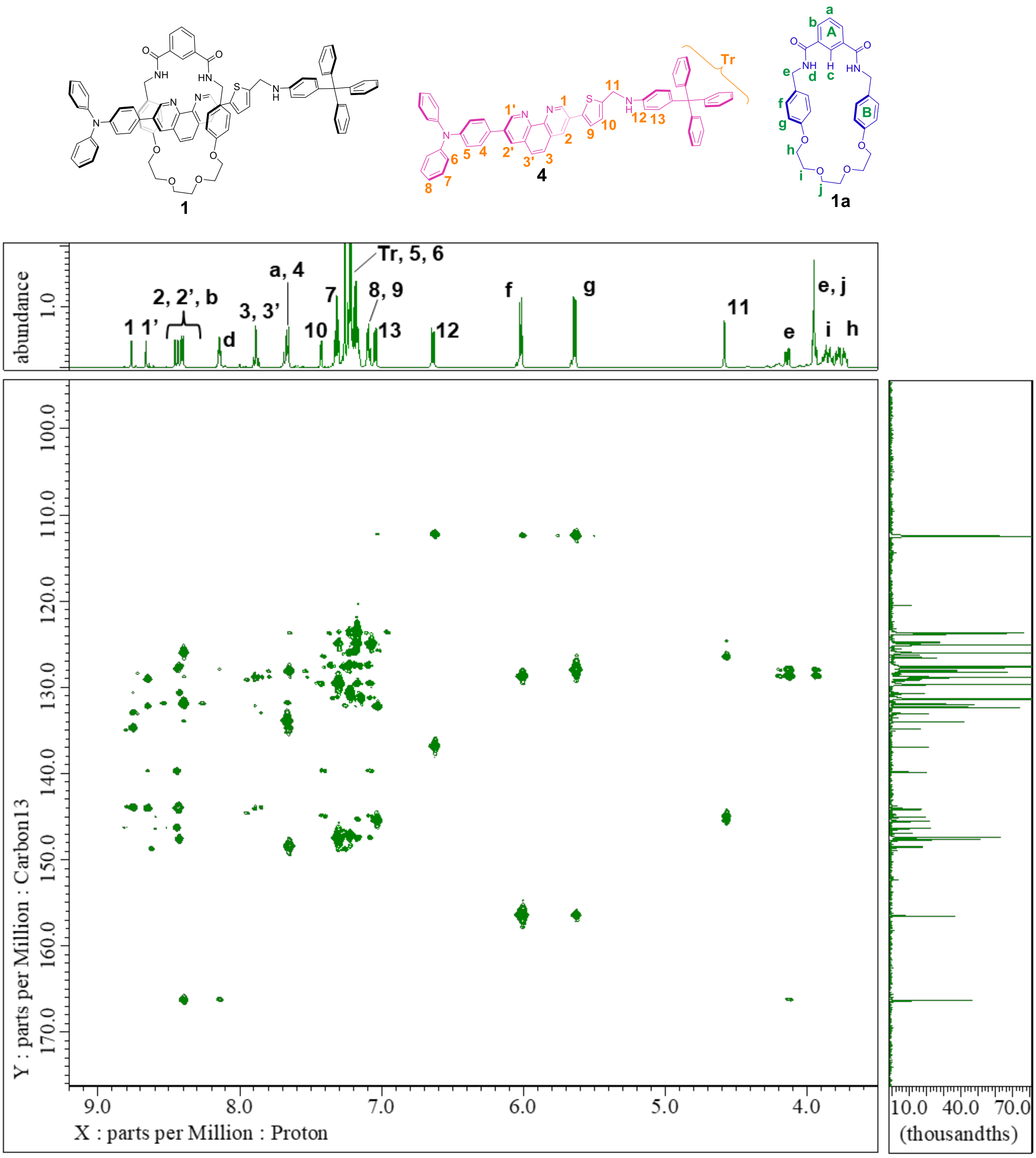

Figure S8-3. HMBC (600 MHz, $CDCl_3$) spectrum of rotaxane **1**.

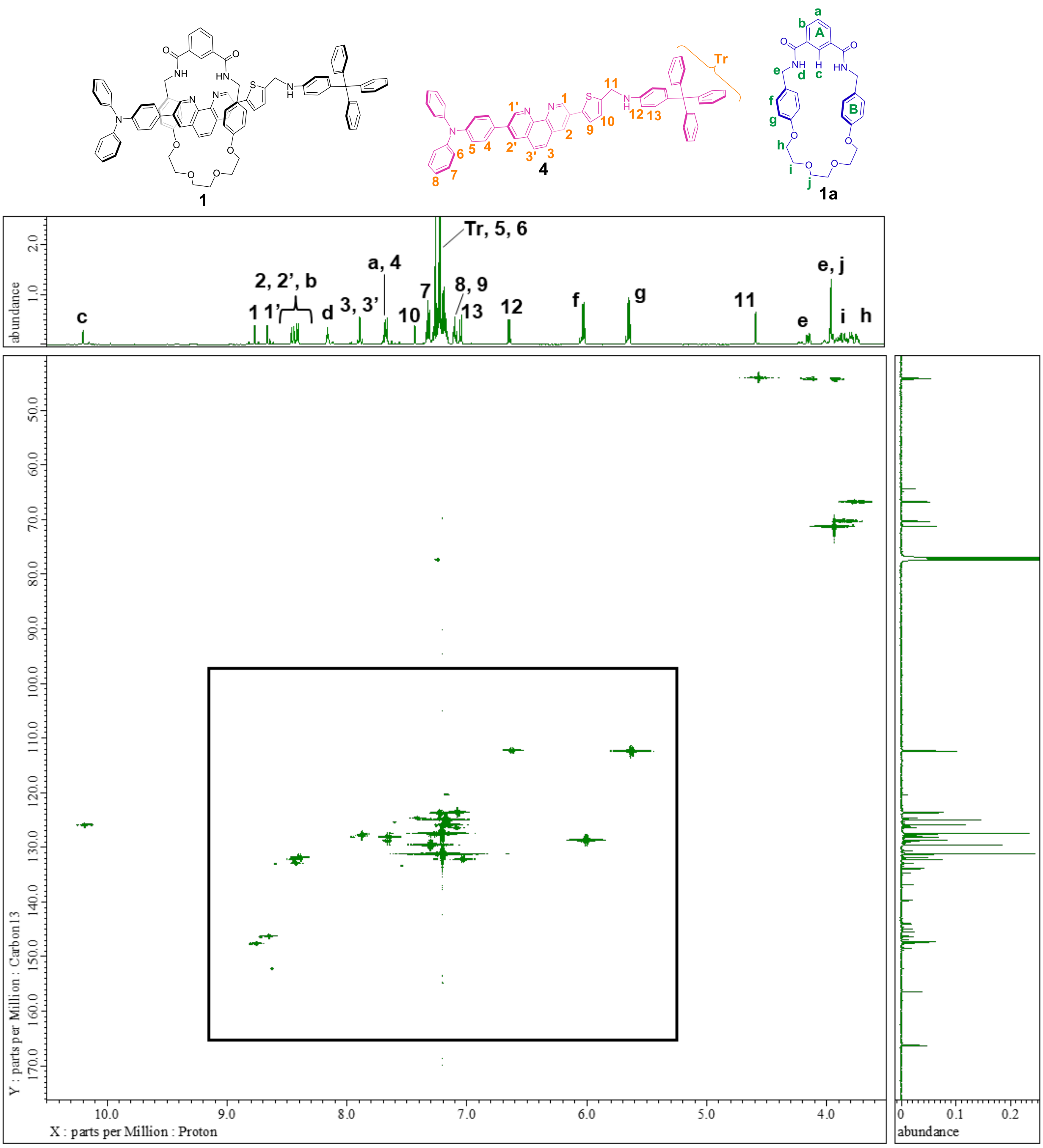

Figure S9-1. HMQC (600 MHz, $CDCl_3$) spectrum of rotaxane **1**.

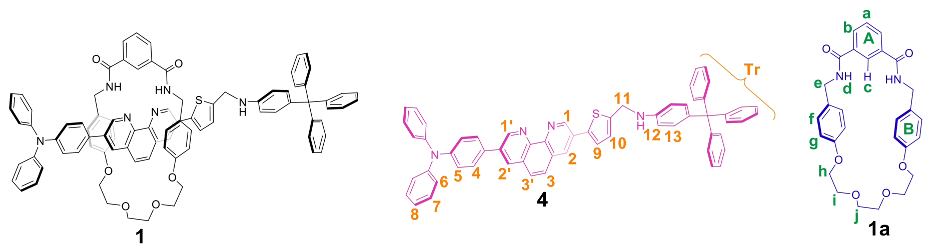

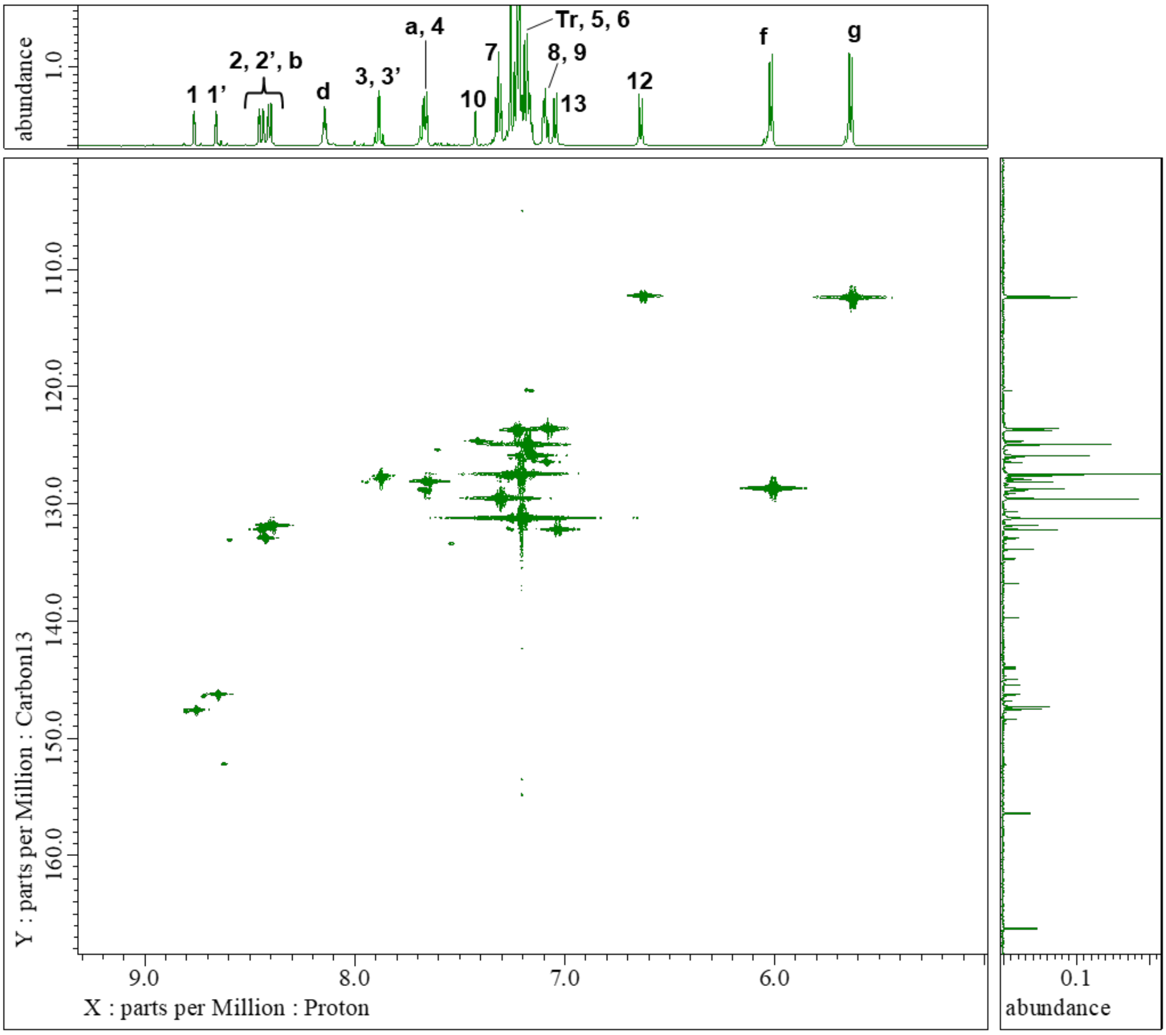

Figure S9-2. HMQC (600 MHz, $CDCl_3$) spectrum of rotaxane **1**.

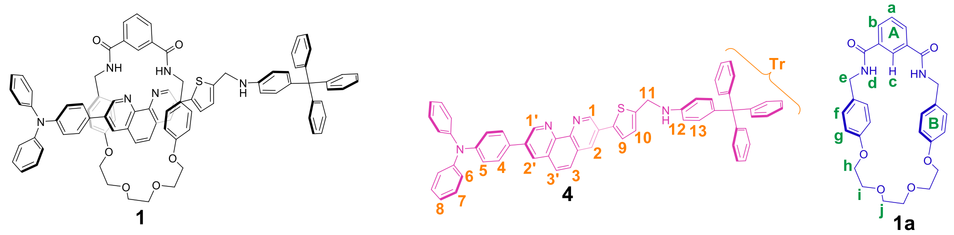

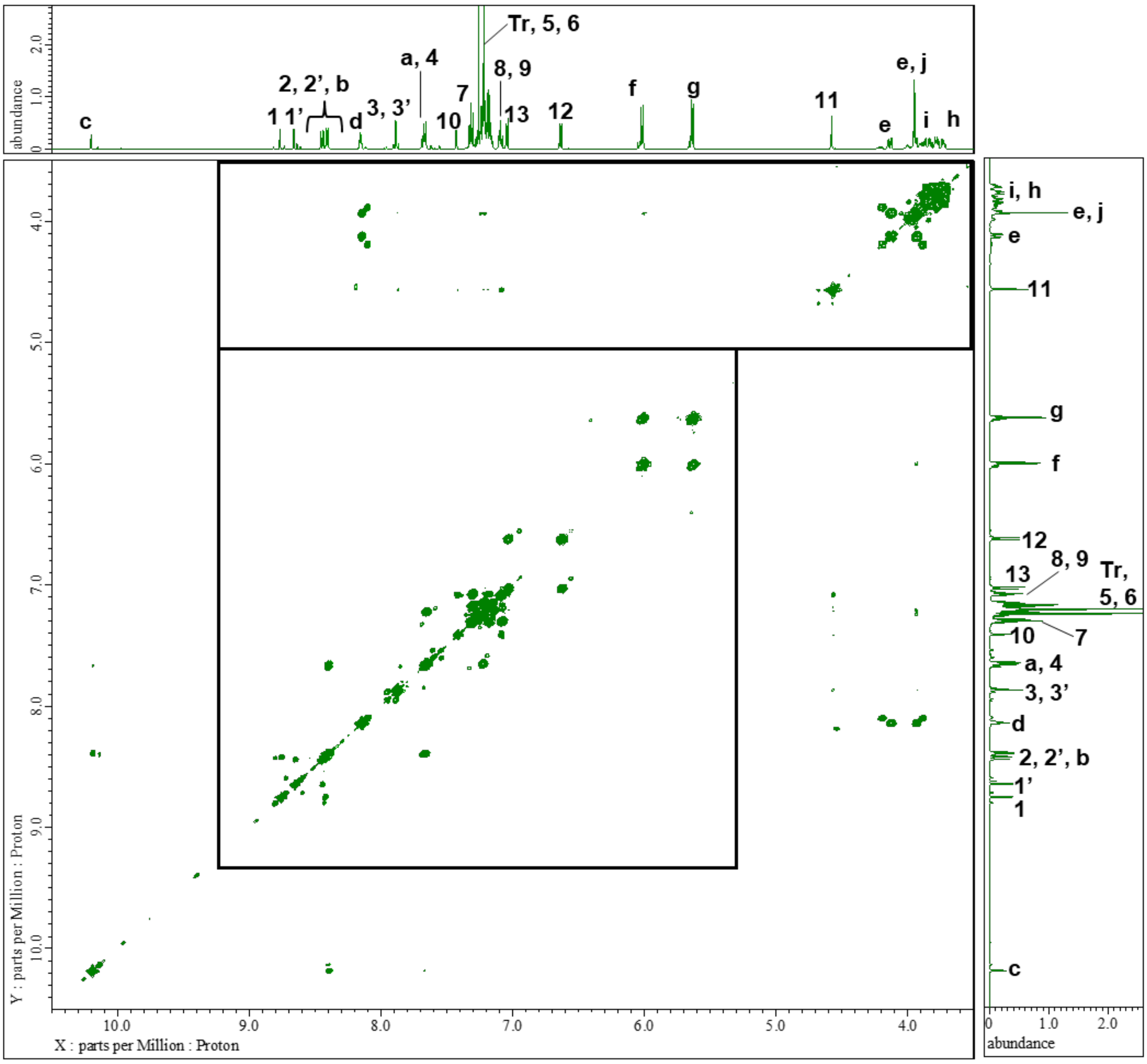

Figure S10-1. ^{1}H -^{1}H COSY (600 MHz, $CDCl_3$) spectrum of rotaxane **1**.

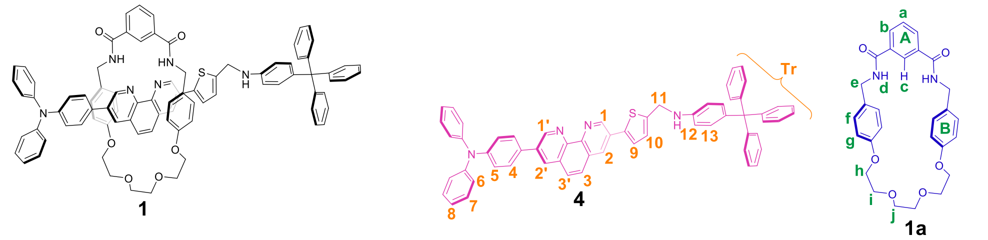

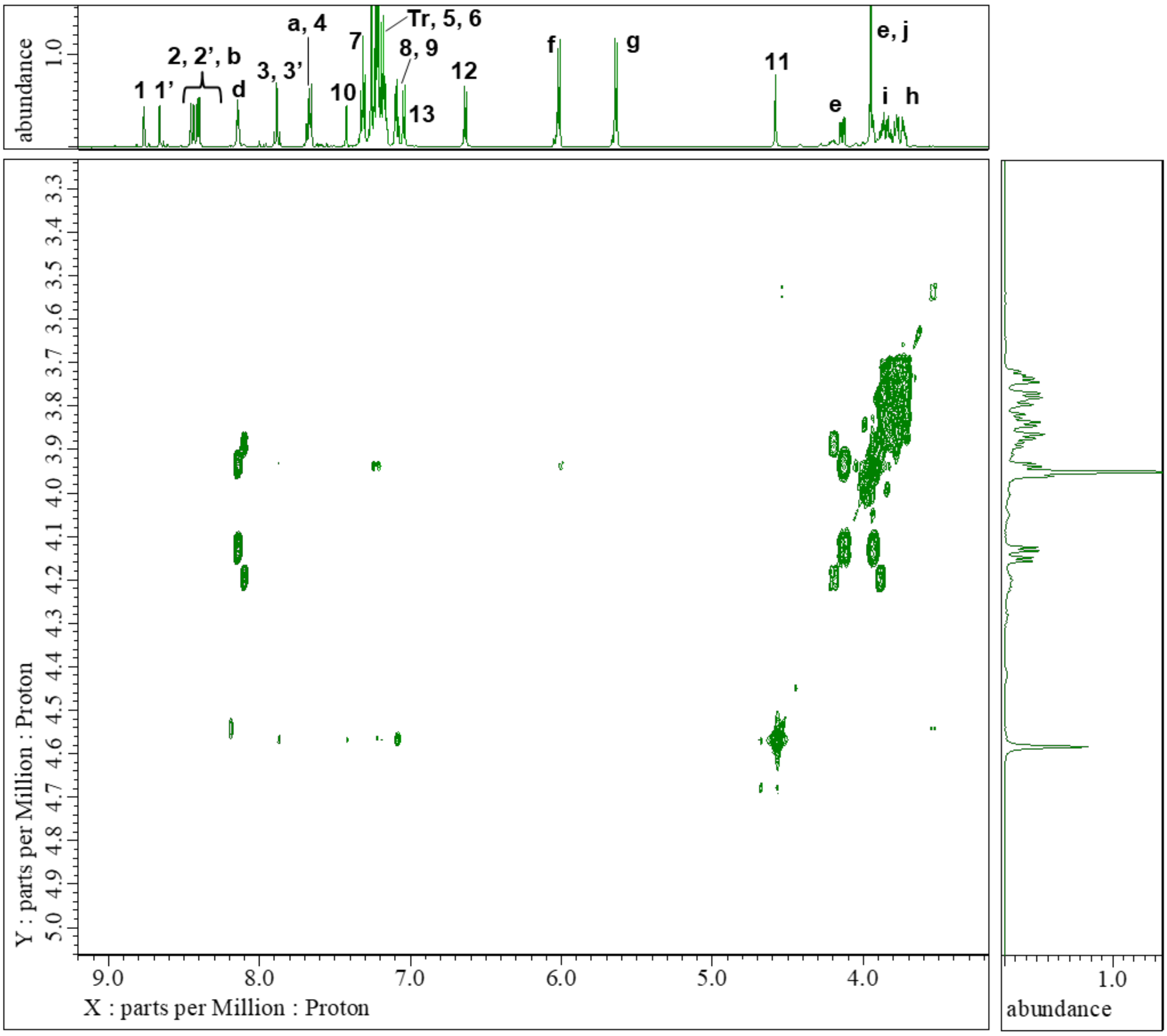

Figure S10-2. ^{1}H -^{1}H COSY (600 MHz, $CDCl_3$) spectrum of rotaxane **1**.

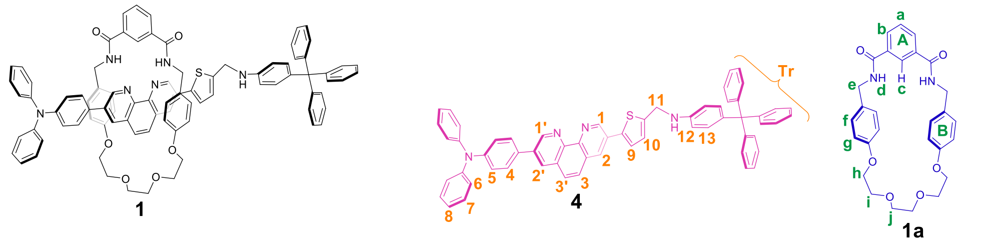

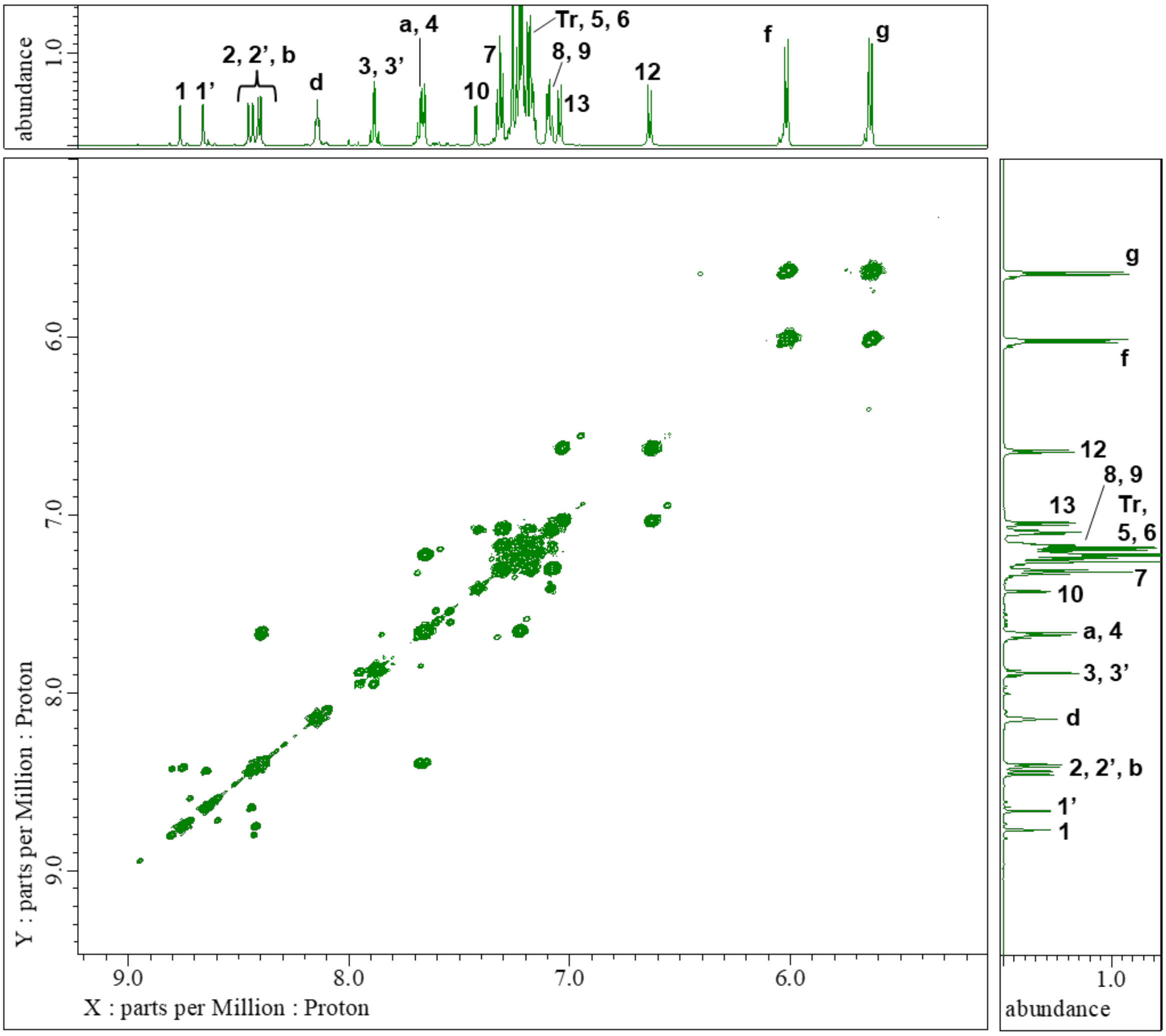

Figure S10-3. ^{1}H -^{1}H COSY (600 MHz, $CDCl_3$) spectrum of rotaxane **1**.

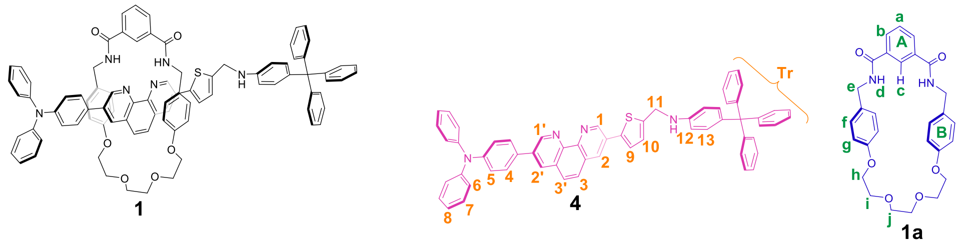

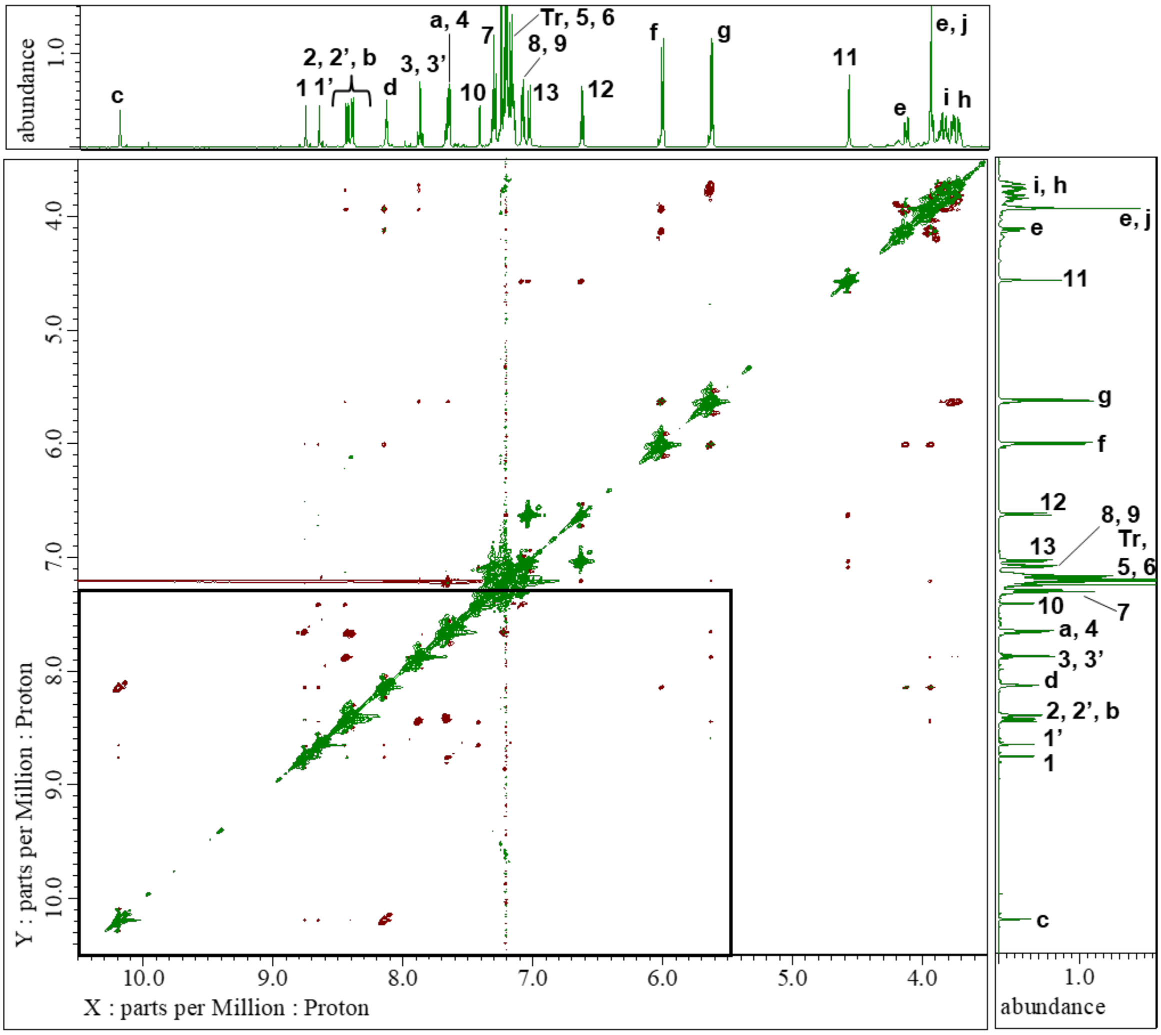

Figure S11-1. ROESY (600 MHz, $CDCl_3$) spectrum of rotaxane **1**.

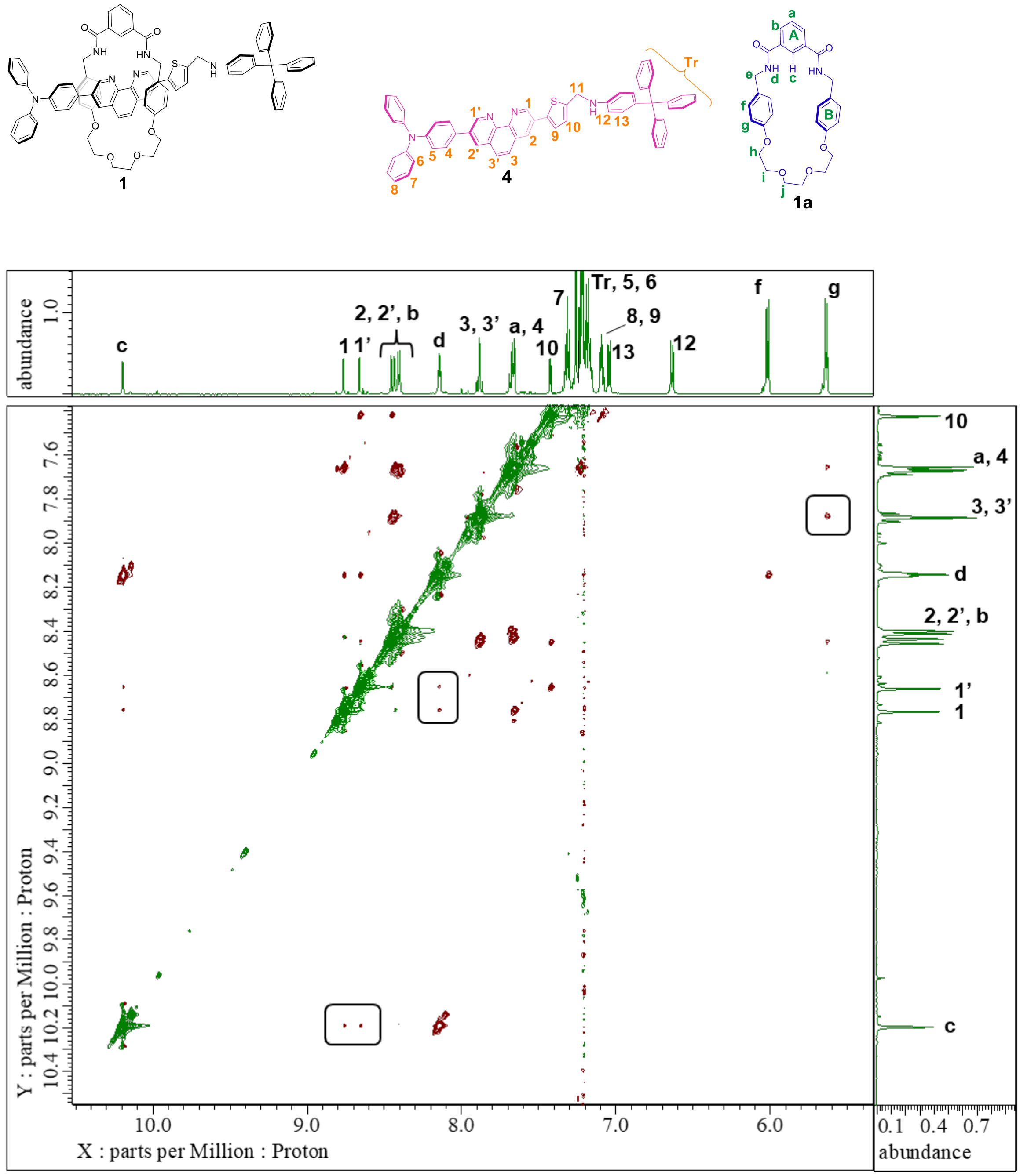

Figure S11-2. ROESY (600 MHz, $CDCl_3$) spectrum of rotaxane **1**.

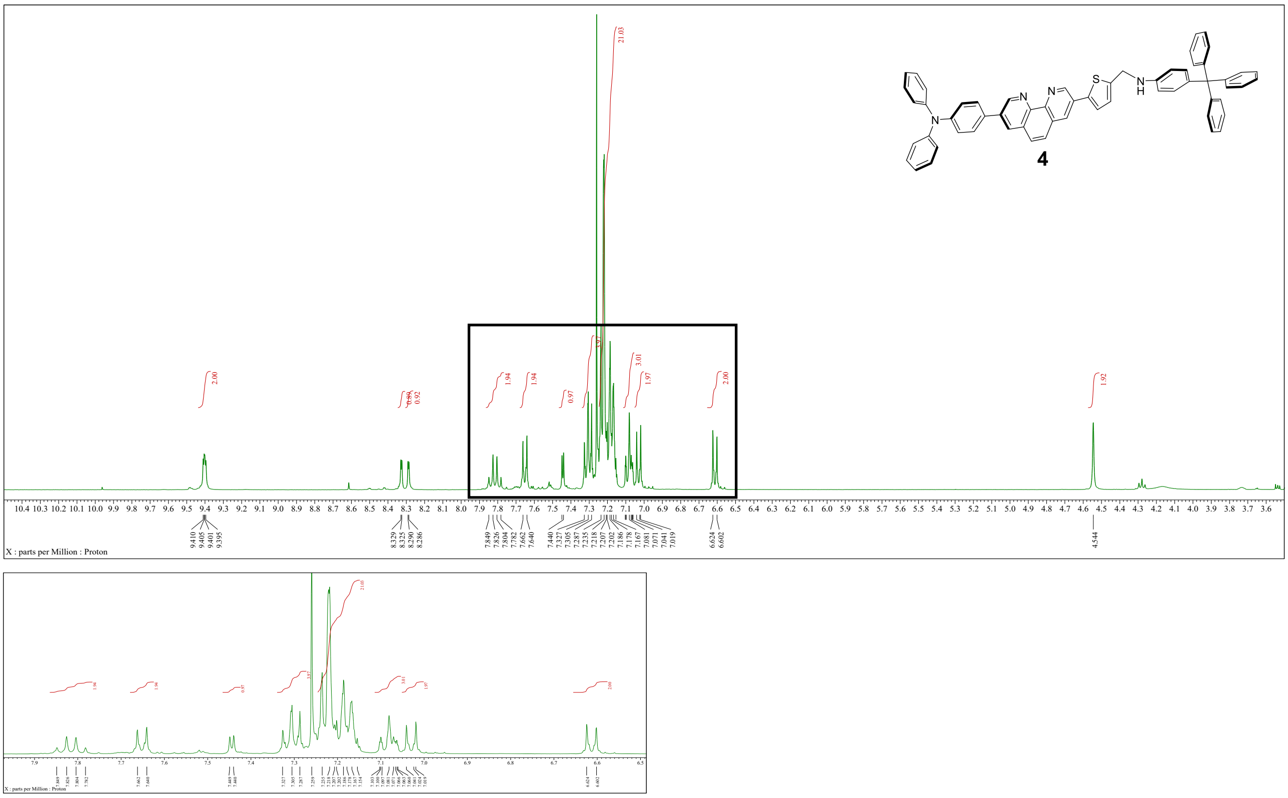

Figure S12. ^{1}H NMR (400 MHz, $CDCl_3$) spectrum of *N,N*-diphenyl-4-(8-(5-(((4-tritylphenyl)amino)methyl)thiophen-2-yl)-1,10-phenanthrolin-3-yl)aniline (**4**).

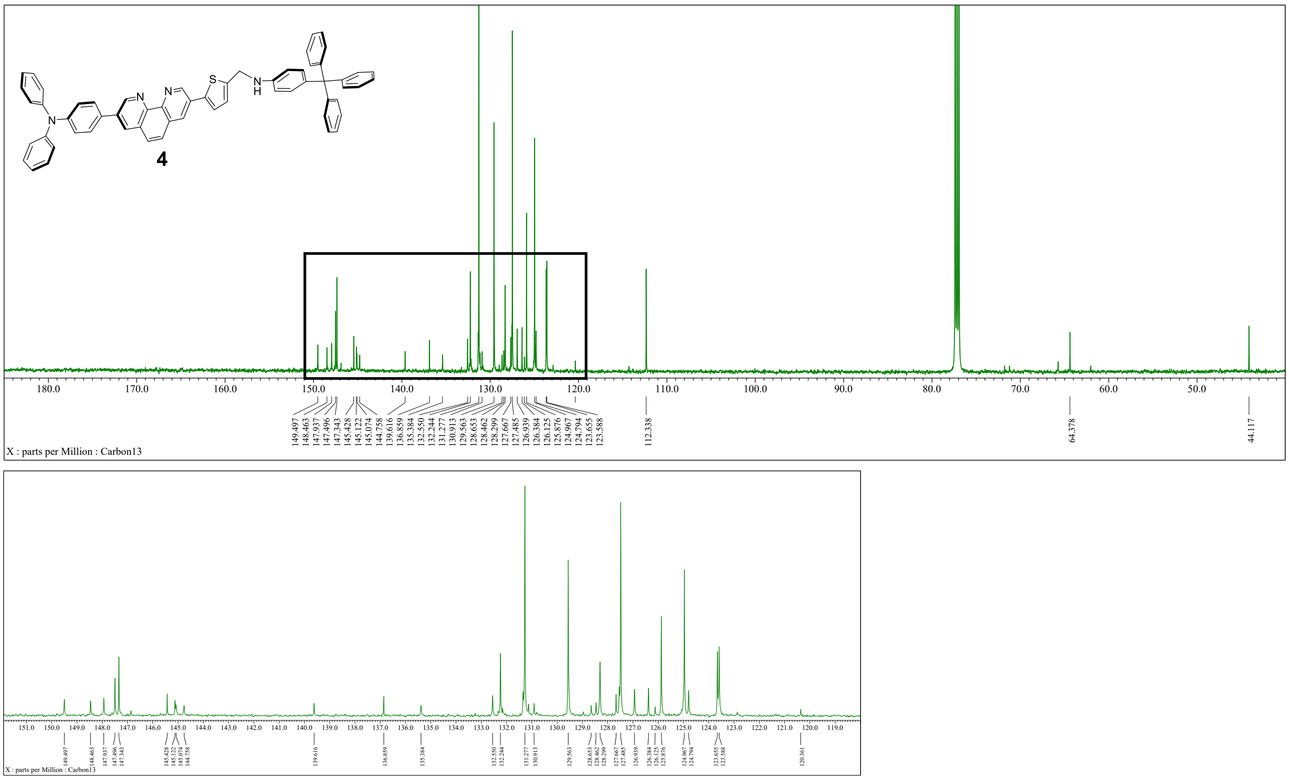

Figure S13. ^{13}C NMR (150 MHz, $CDCl_3$) spectrum of

N,N-diphenyl-4-(8-(5-(((4-tritylphenyl)amino)methyl)thiophen-2-yl)-1,10-phenanthrolin-3-yl)aniline (**4**).

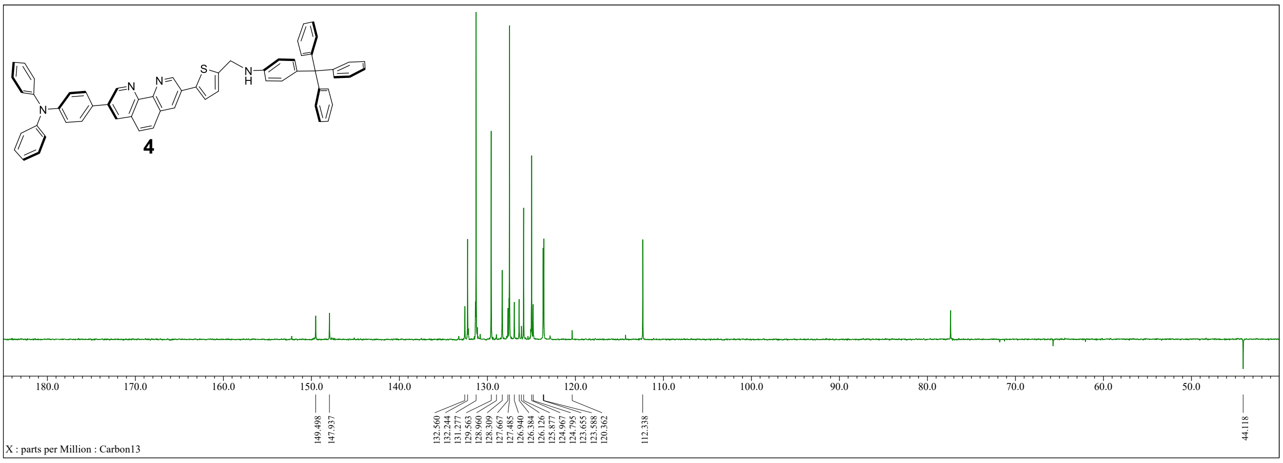

Figure S14. DEPT 135° (150 MHz, $CDCl_3$) spectrum of *N,N*-diphenyl-4-(8-(5-(((4-tritylphenyl)amino)methyl)thiophen-2-yl)-1,10-phenanthrolin-3-yl)aniline (**4**).

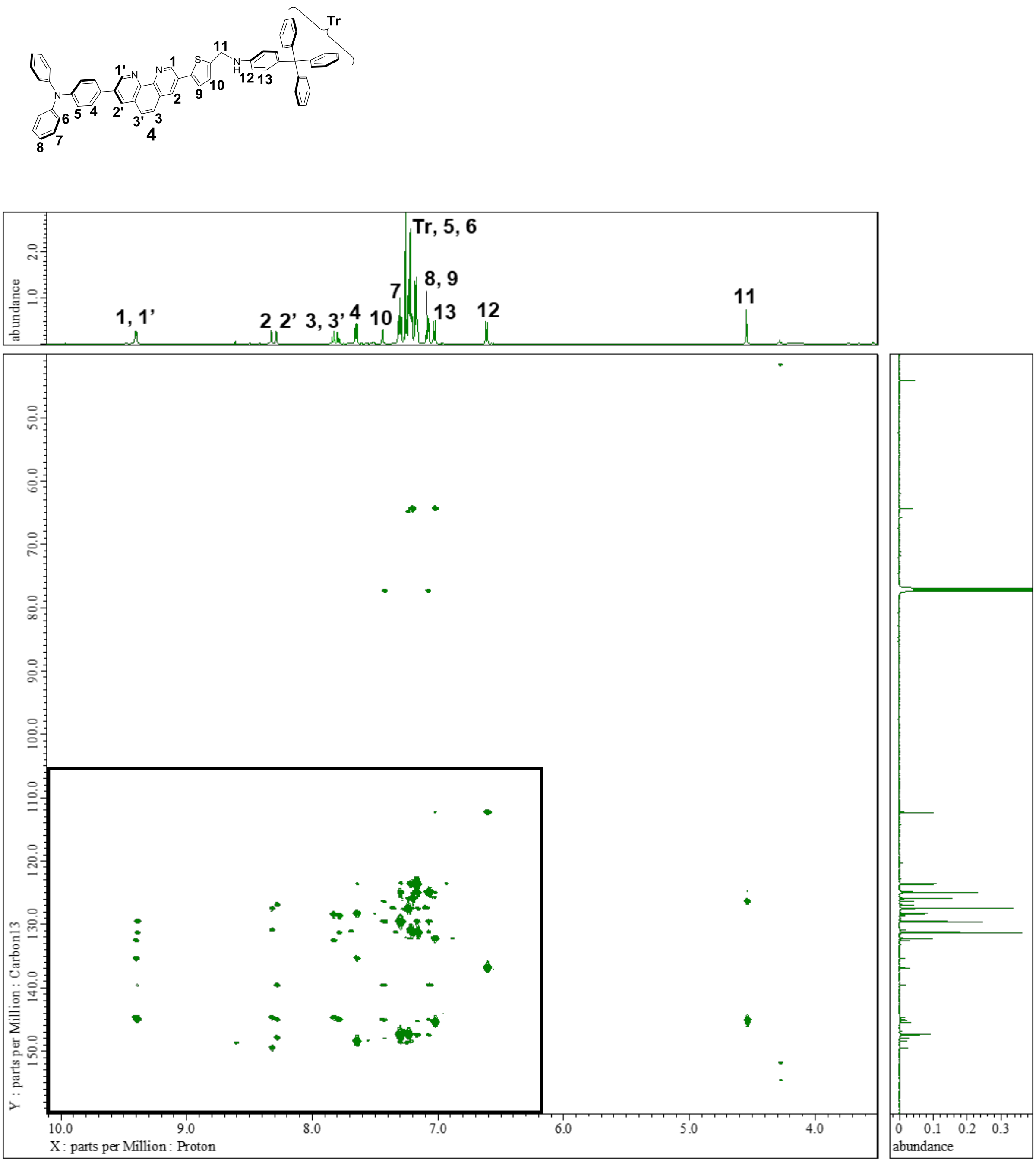

Figure S15-1. HMBC (600 MHz, $CDCl_3$) spectrum of *N,N*-diphenyl-4-(8-(5-(((4-tritylphenyl)amino)methyl)thiophen-2-yl)-1,10-phenanthrolin-3-yl)aniline (**4**).

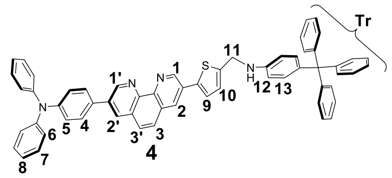

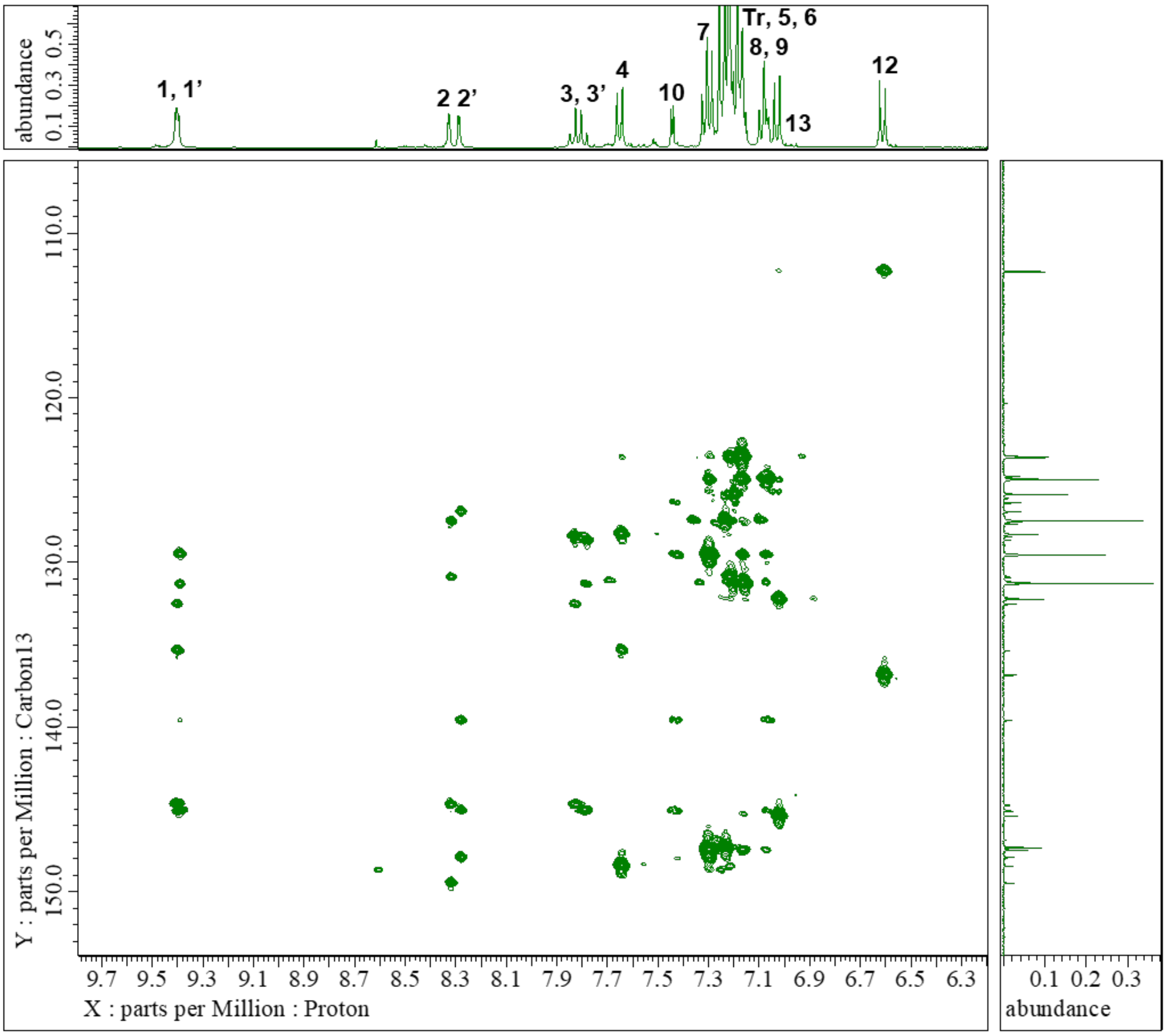

Figure S15-2. HMBC (600 MHz, $CDCl_3$) spectrum of *N,N*-diphenyl-4-(8-(5-(((4-tritylphenyl)amino)methyl)thiophen-2-yl)-1,10-phenanthrolin-3-yl)aniline (**4**).

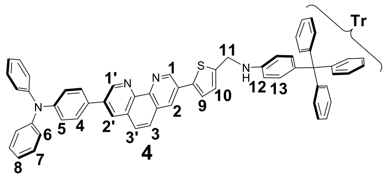

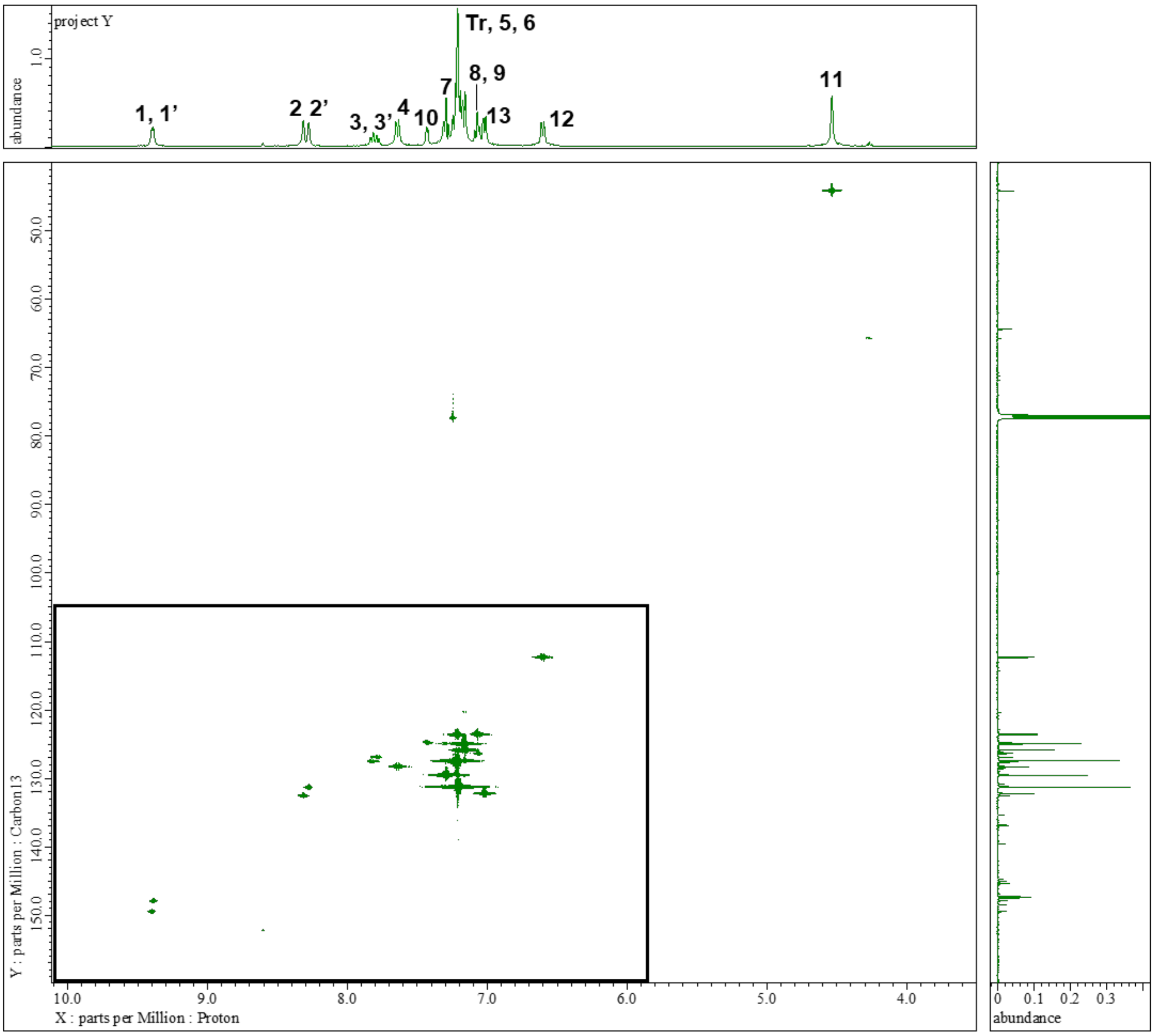

Figure S16-1. HMQC (600 MHz, $CDCl_3$) spectrum of *N,N*-diphenyl-4-(8-(5-(((4-tritylphenyl)amino)methyl)thiophen-2-yl)-1,10-phenanthrolin-3-yl)aniline (**4**).

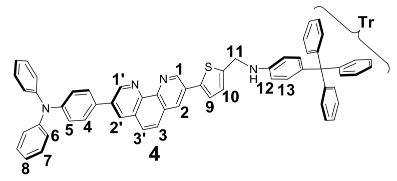

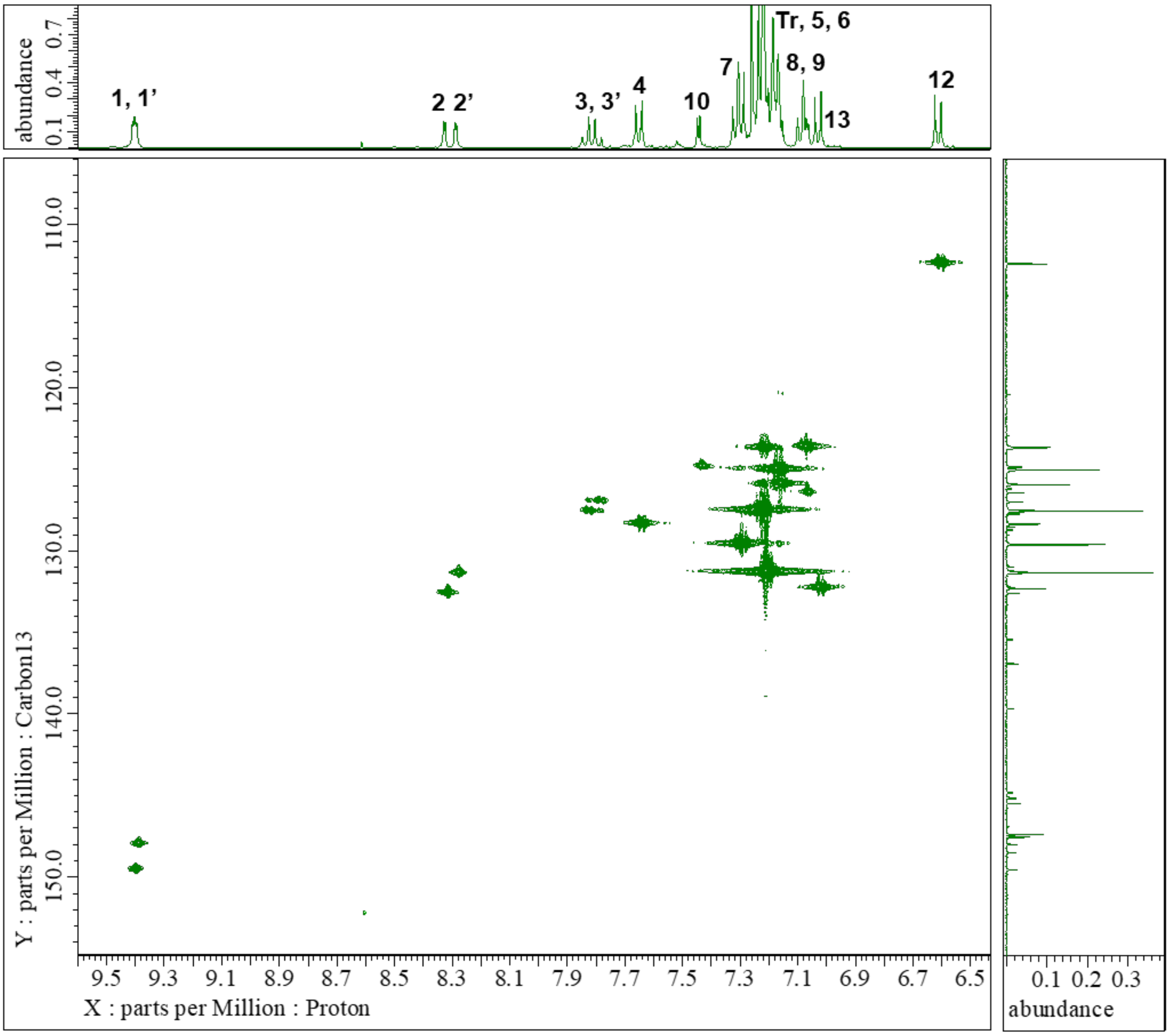

Figure S16-2. HMQC (600 MHz, $CDCl_3$) spectrum of *N,N*-diphenyl-4-(8-(5-(((4-tritylphenyl)amino)methyl)thiophen-2-yl)-1,10-phenanthrolin-3-yl)aniline (**4**).

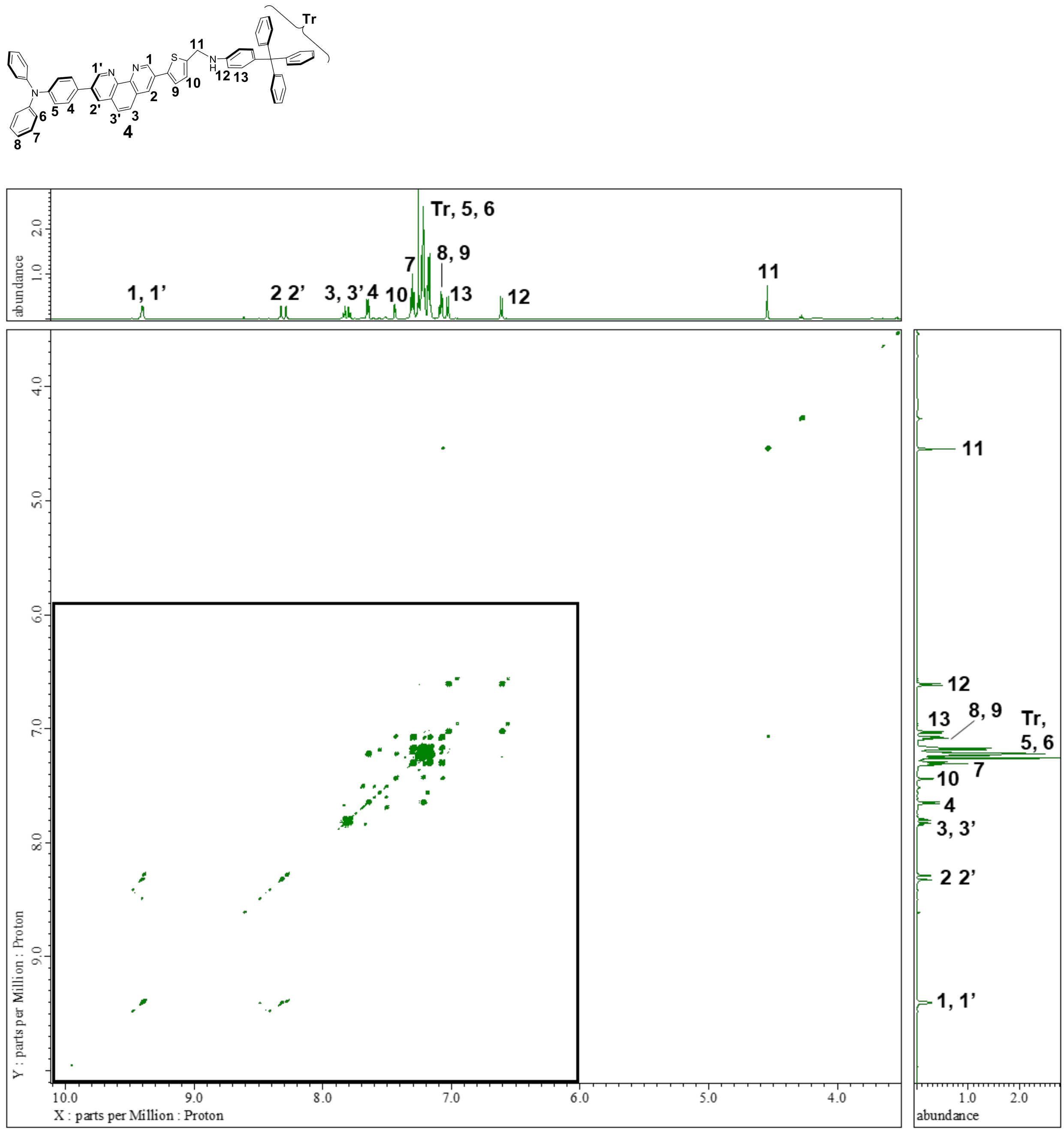

Figure S17-1. ^{1}H-^{1}H COSY (600 MHz, $CDCl_3$) spectrum of *N,N*-diphenyl-4-(8-(5-(((4-tritylphenyl)amino)methyl)thiophen-2-yl)-1,10-phenanthrolin-3-yl)aniline (**4**).

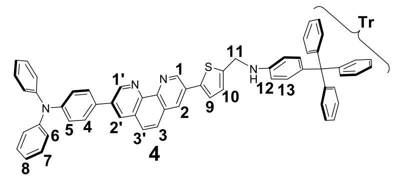

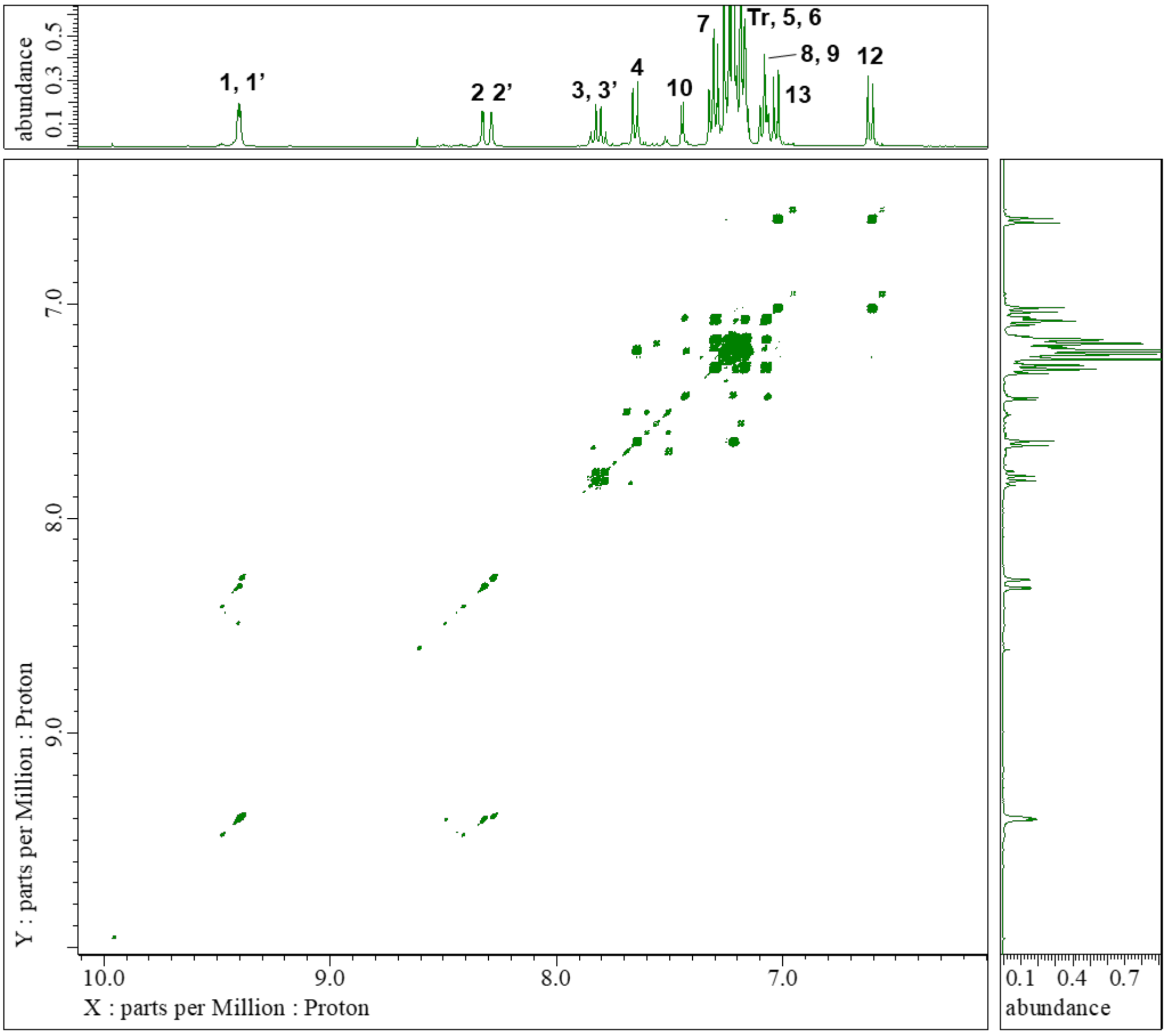

Figure S17-2. ^{1}H-^{1}H COSY (600 MHz, $CDCl_3$) spectrum of *N,N*-diphenyl-4-(8-(5-(((4-tritylphenyl)amino)methyl)thiophen-2-yl)-1,10-phenanthrolin-3-yl)aniline (**4**).

4. Mass spectrum

(a)

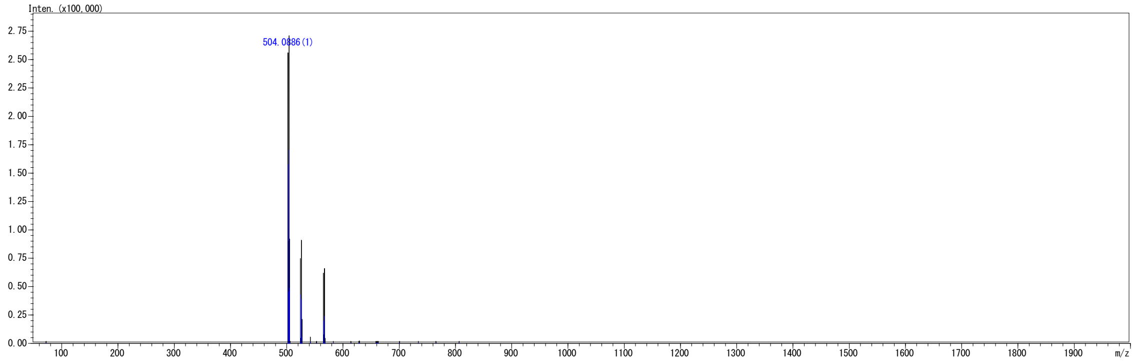

(b)

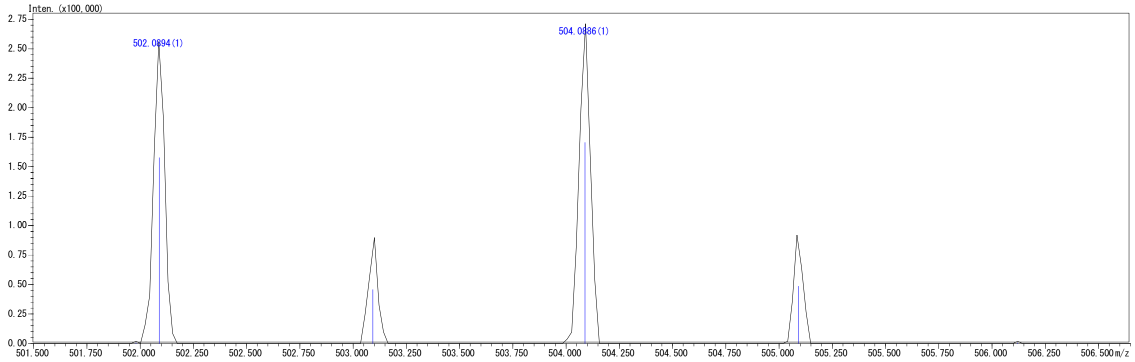

(c)

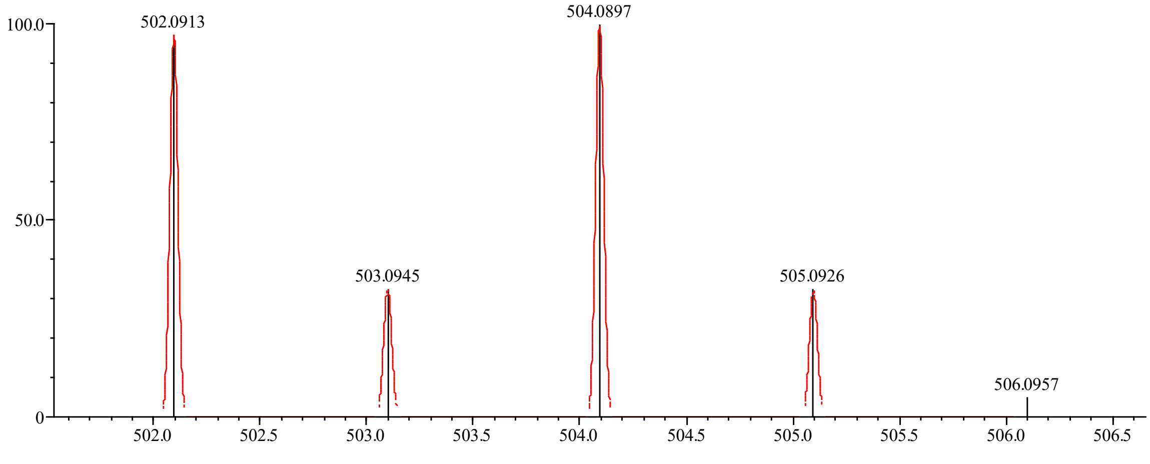

Figure S18. IT-TOF MS of 4-(8-Bromo-1,10-phenanthrolin-3-yl)-*N,N*-diphenylaniline (**2**).

(a) Full mass range spectrum, (b) Expanded mass range spectrum, and (c) calculated mass spectrum.

(a)

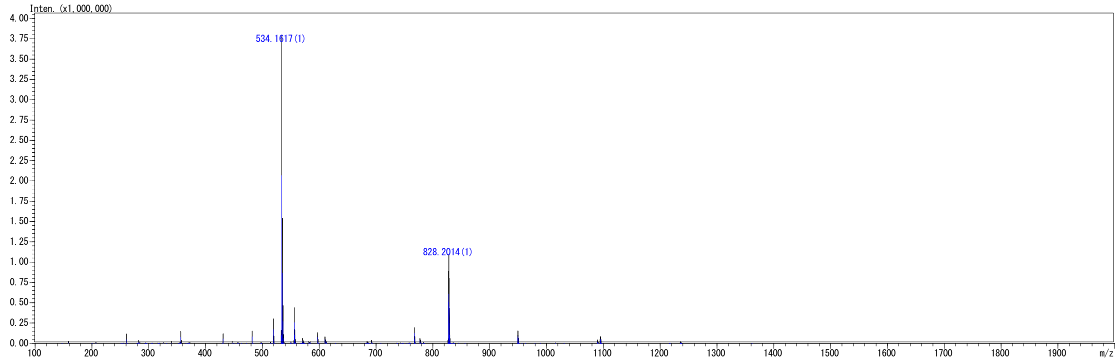

(b)

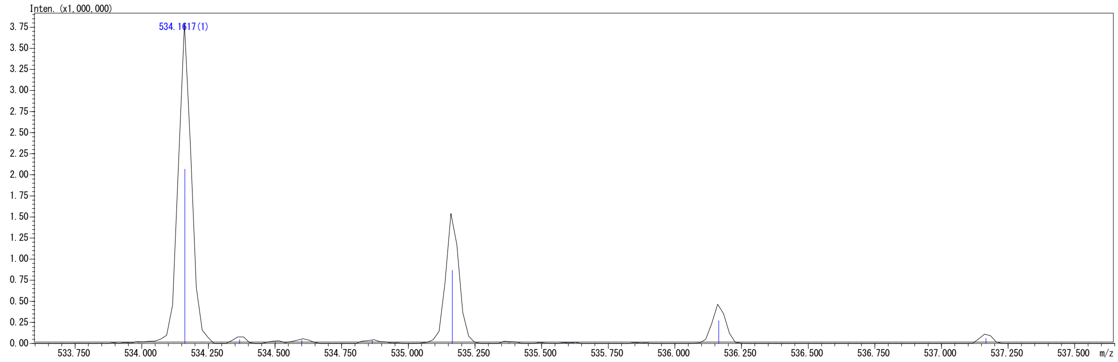

(c)

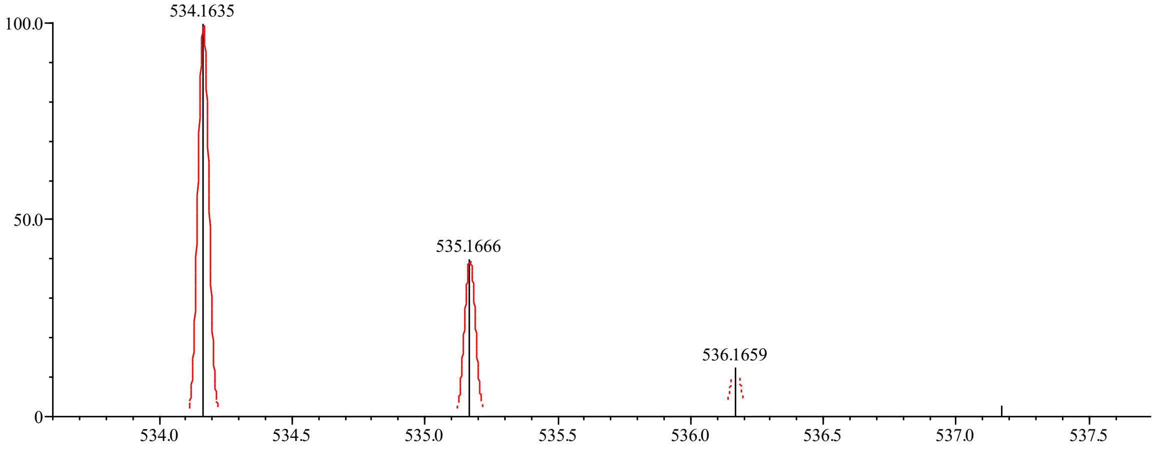

Figure S19. IT-TOF MS of 5-(8-(4-(diphenylamino)phenyl)-1,10-phenanthrolin-3-yl)thiophene-2-carbaldehyde (**3**). (a) Full mass range spectrum, (b) Expanded mass range spectrum, and (c) calculated mass spectrum.

(a)

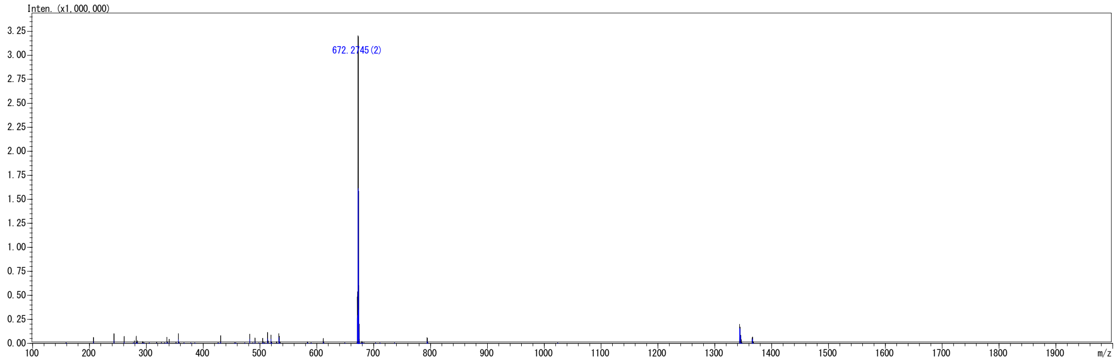

(b)

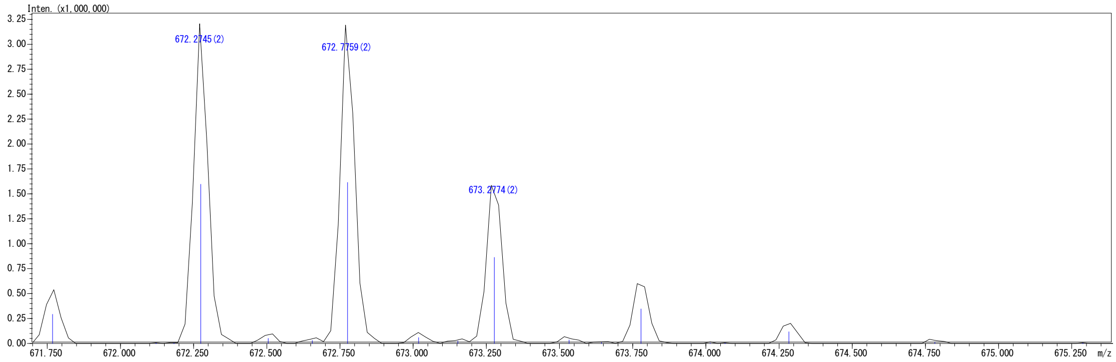

(c)

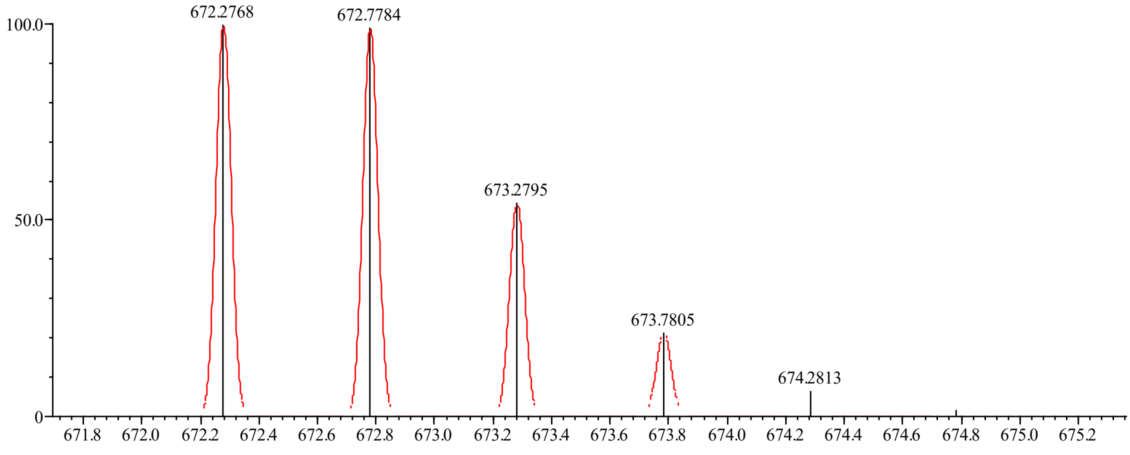

Figure S20. IT-TOF MS of rotaxane **1**.

(a) Full mass range spectrum, (b) Expanded mass range spectrum, and (c) calculated mass spectrum.

(a)

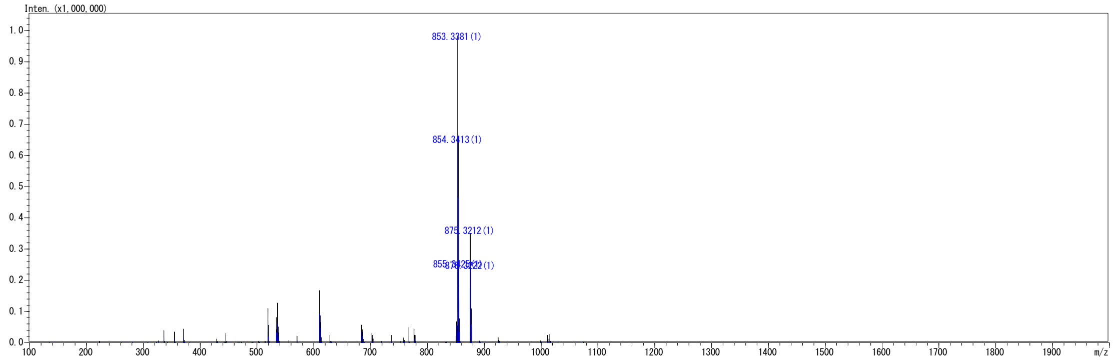

(b)

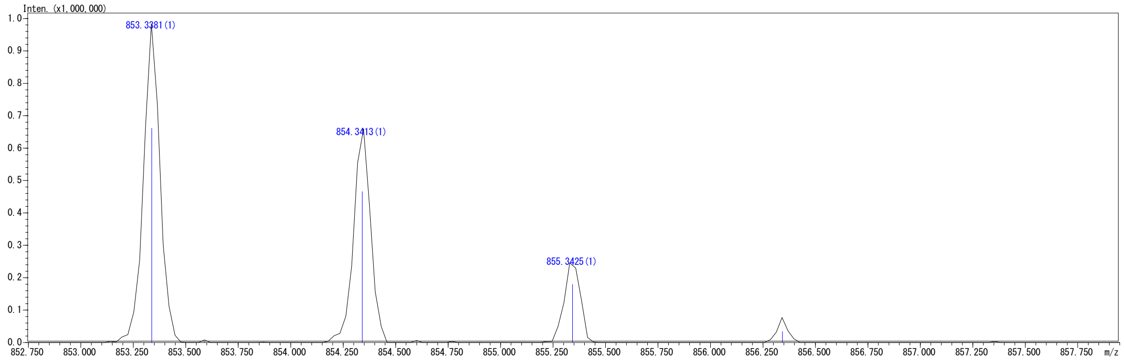

(c)

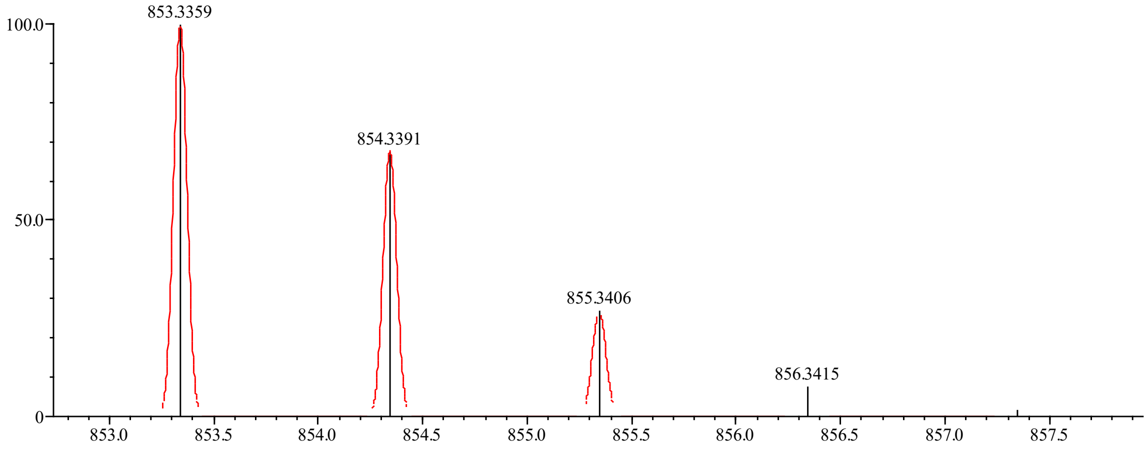

Figure S21. IT-TOF MS of *N,N*-diphenyl-4-(8-(5-(((4-tritylphenyl)amino)methyl)thiophen-2-yl)-1,10-phenanthrolin-3-yl)aniline (**4**).

(a) Full mass range spectrum, (b) Expanded mass range spectrum, and (c) calculated mass spectrum.

5. ^{1}H NMR Titrations and Binding Constants

Titration result was acquired by a JEOL JNM-ECZ400s 400 MHz NMR spectrometer and performed with the starting concentration of host **1a** at 6.2 mM and appropriate aliquots of guests **3** at 138 mM solution with a microsyringe. The protons (f and g) for the host **1a** were followed during the course of the titration. The complexation equilibrium was fast on the NMR time scale and gave signals at weight averaged chemical shifts of the free and complexed host. The binding constants (K_a) for the complexation were obtained based on the chemical shift change by the titration experiment followed by non-linear least-square data treatment method with 95% confidence interval applied by Student's t-distribution reported by Hirose.[S1] The volume change for the titrated solutions was properly accounted for in the Hirose's method. The limiting chemical shifts, Δ_0, which mean the difference in δ values for the protons of the host **1a** in the uncomplexed and fully complexed species, and the standard deviations as the curve fitting errors for the association constants were determined by SOLVSTAT.[S2] The NMR titration curves are shown below.

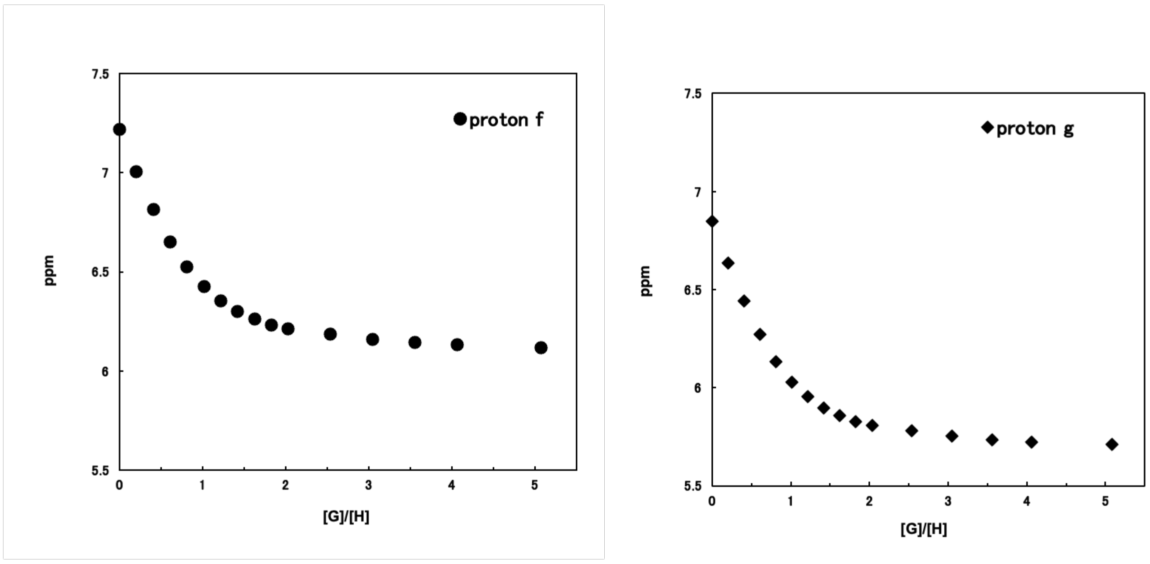

Figure S22. NMR titration curves for **3** with macrocycle **1a** monitoring the protons f and g in $CDCl_3$ at 293 K.

6. ^{1}H NMR Studies for Molecular Switching of [2]Rotaxane 1 by Acid and Base

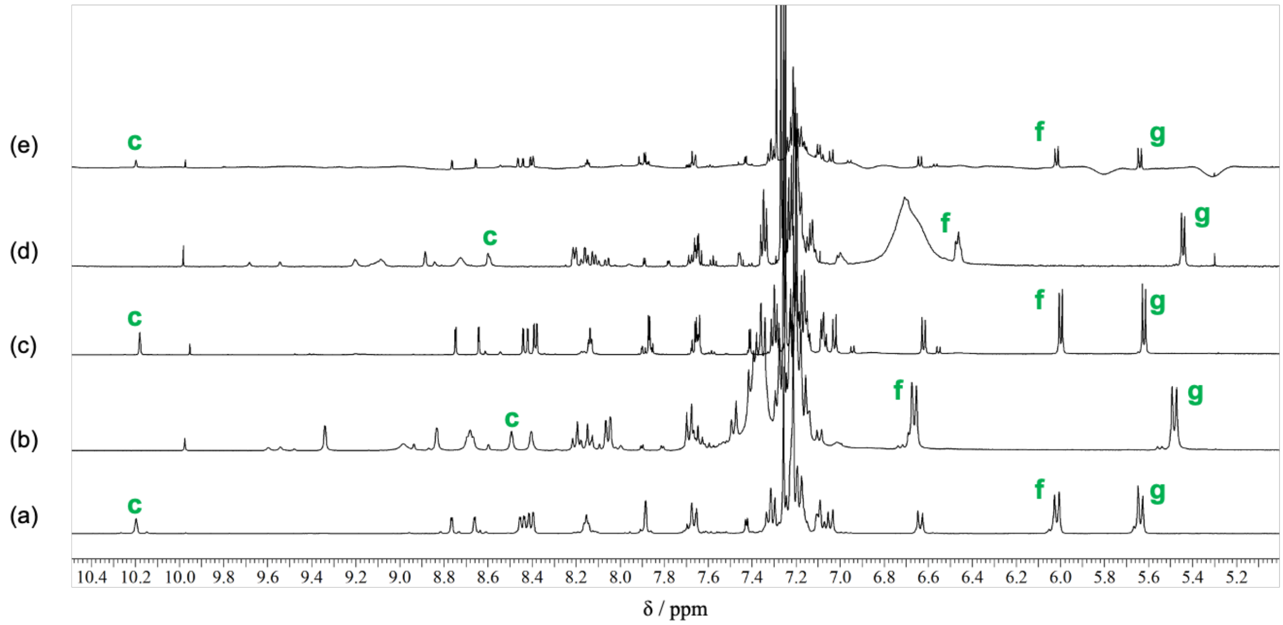

Figure S23. Partial ^{1}H NMR spectra (400 MHz, $CDCl_3$) of (a) rotaxane **1**, (b) the mixture obtained after adding TFA (9 eq.) to the rotaxane **1** solution, (c) the mixture obtained after adding NEt_3 (9 eq.) to the mixture obtained in (b), (d) the mixture obtained after adding TFA (10 eq.) to the mixture obtained in (c), and (e) the mixture obtained after adding NEt_3 (19 eq.) to the mixture obtained in (d).

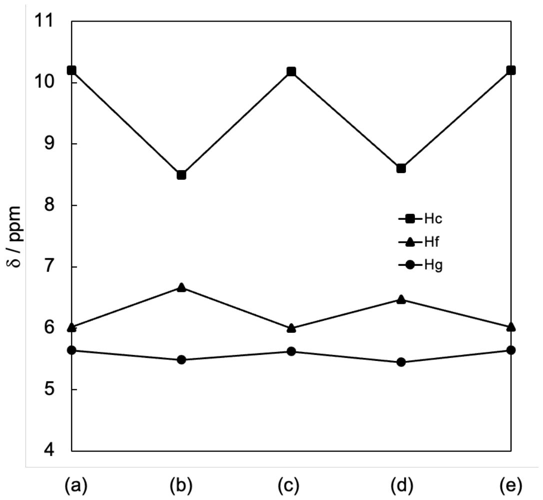

Figure S24. Chemical shift changes of protons c, f, and g on rotaxane **1** in partial ^{1}H NMR spectra shown in Figure S23 (a)-(e). [(a) Rotaxane 1. First cycle; (b) addition of TFA and (c) then addition of NEt_3. Second cycle; (d) addition of TFA and (e) then addition of NEt_3.]

7. References

S1 K. Hirose, *J. Incl. Phenom. Macrocycl. Chem*. 2001, **39**, 193-209.

S2 E. J. Billo, *Excel for Chemists 2nd Ed*., Wiley, New York, 2001.
